# Lysosome-centered nanomedicine for cancer therapy: mechanisms, materials, and modalities

**DOI:** 10.1186/s12951-026-04438-7

**Published:** 2026-04-14

**Authors:** Rongwei Xu, Nina Li, Xu Chen, Meiyan Zou, Weiyao Feng, Zihao Zhou, Zhiyu Guo, Xinyuan Zhao, Shuguang Liu, Li Cui

**Affiliations:** 1 Stomatological Hospital, School of Stomatology, Southern Medical University, Guangzhou, 510280 Guangdong China; 2 School of Dentistry, University of California, Los Angeles, Los Angeles, CA 90095 USA

**Keywords:** Lysosome, Nanomedicine, Cancer, Drug delivery, Targeted therapy, LYTAC

## Abstract

The lysosome, long regarded as a terminal degradative organelle, has emerged as a central and programmable hub for anticancer nanomedicine. Advances in materials science, chemical biology, and intracellular trafficking have enabled nanomaterials to exploit lysosomal pathways not only for drug degradation but also for controlled payload activation, cytosolic delivery, signaling modulation, and organelle-confined therapeutic execution. In this review, we present a unified framework for lysosome-centered cancer nanotherapy organized along five complementary design axes: receptor-guided lysosomal protein degradation platforms such as lysosome-targeting chimeras (LYTACs); lysosome-triggered payload activation and release; endolysosomal escape strategies enabling cytosolic or nuclear delivery; nanomaterial-mediated modulation of lysosomal function and autophagic flux; and lysosome-focused therapeutic modalities driven by external stimuli or catalytic reactions, including photodynamic, sonodynamic, photothermal, and chemodynamic therapies. Across these paradigms, the lysosome functions not merely as a passive sink but as an active determinant of therapeutic specificity, efficacy, and immune consequence. We discuss how rational nanomaterial design leverages lysosomal acidity, enzymatic activity, membrane vulnerability, and signaling roles to overcome drug resistance, reprogram tumor immunity, and enable previously inaccessible therapeutic interventions. Finally, we highlight key translational challenges—including receptor heterogeneity, material complexity, biological feedback, and manufacturing scalability—and outline future directions toward clinically viable, precision lysosome-targeted nanomedicines.

**Graphical Abstract:**

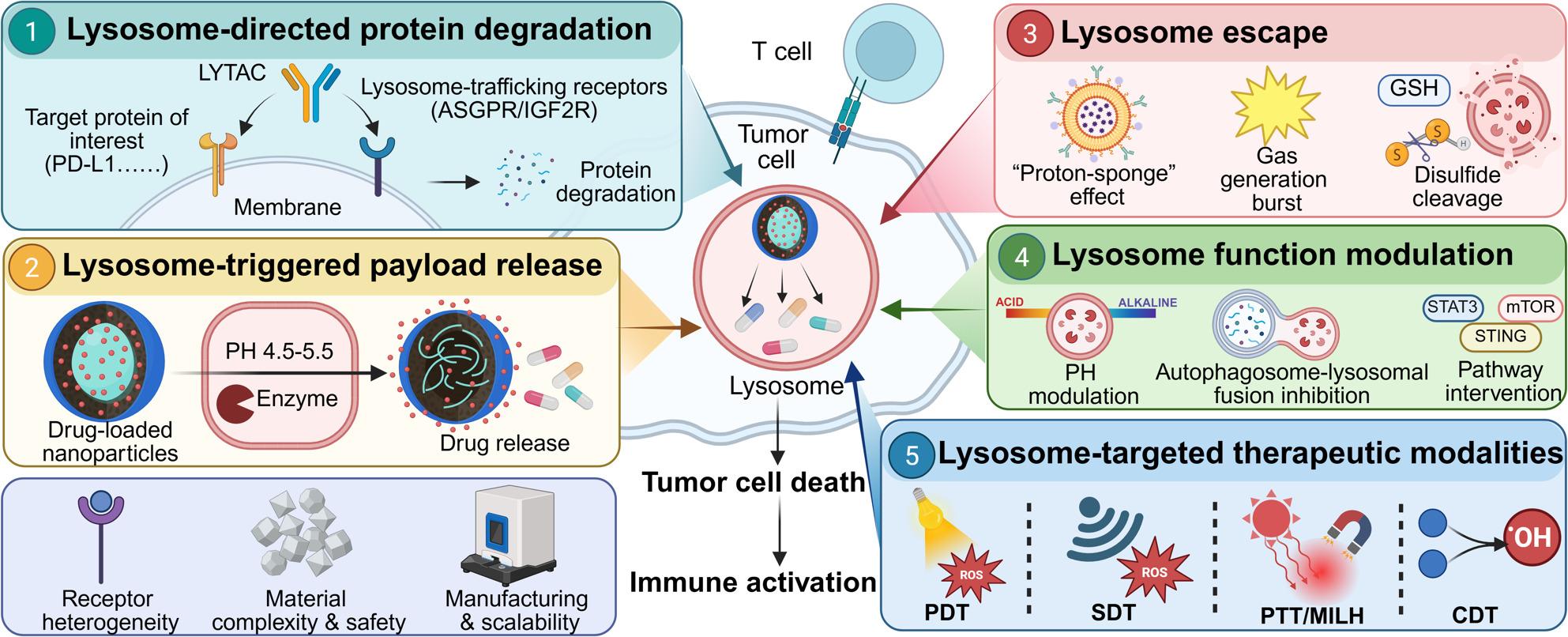

## Introduction

Cancer remains one of the most formidable public health challenges worldwide. According to the most recent Global Burden of Disease analysis published in The Lancet (2025), an estimated 18.5 million new cancer cases (excluding non-melanoma skin cancer) were diagnosed globally in 2023, resulting in approximately 10.4 million deaths. Projections further indicate that by 2050, annual cancer incidence will rise to 30.5 million cases—representing a 60.7% increase relative to 2024—while cancer-associated mortality is expected to reach 18.6 million deaths, an increase of 74.5% [1]. This sustained escalation in both incidence and mortality underscores an urgent need for more effective and durable therapeutic strategies.

For decades, surgery, chemotherapy, and radiotherapy have constituted the mainstays of cancer treatment. Despite their clinical value, these conventional modalities suffer from intrinsic limitations. Chemotherapy and radiotherapy frequently exhibit inadequate tissue penetration, limited tumor selectivity, and pronounced off-target toxicity, leading to severe systemic side effects and compromised quality of life [2]. Moreover, cancer cells readily acquire resistance through multiple adaptive mechanisms, including enhanced drug efflux, activation of DNA repair pathways, evasion of apoptosis, and metabolic rewiring [3, 4]. Even after initial responses, tumor recurrence remains common, reflecting the inability of existing therapies to eradicate heterogeneous and adaptable malignant cell populations.

To overcome these limitations, nanotechnology has emerged as a transformative approach in cancer therapy. Nanomaterials—typically ranging from 1 to 100 nm in size—possess unique physicochemical properties, including high surface-to-volume ratios and engineerable interfaces, which enable unprecedented control over drug delivery [5]. Through the enhanced permeability and retention (EPR) effect, nanoparticles can preferentially accumulate within tumor tissues, while surface functionalization with targeting ligands such as antibodies, peptides, or aptamers enables selective cellular recognition and reduced systemic toxicity [6]. Importantly, nanomedicine has evolved beyond passive drug carriers toward intelligent, stimulus-responsive platforms. pH-, enzyme-, redox-, or light-responsive nanomaterials can achieve spatiotemporally controlled drug release within the tumor microenvironment (TME) [7–10]. In parallel, nanomaterials themselves can serve as therapeutic agents, exemplified by gold nanostructures for photothermal therapy (PTT) and sulfur- or metal-based nanocomposites for chemodynamic therapy (CDT) [11, 12]. Collectively, these advances have expanded the therapeutic landscape to include immunomodulation, gene regulation, and physical modalities such as photothermal and sonodynamic therapies, offering new avenues to address resistance and metastasis.

Within this rapidly evolving field, the lysosome has emerged as a central intracellular target for cancer nanomedicine. As membrane-bound acidic organelles enriched with hydrolytic enzymes, lysosomes function as the terminal degradative hub for endocytosed materials, damaged organelles, and macromolecules, thereby maintaining cellular homeostasis [13]. In cancer biology, lysosomes play a paradoxical dual role. On one hand, they support tumor survival by sustaining protective autophagy under metabolic stress and by mediating the recycling of key membrane proteins—such as PD-L1 and epidermal growth factor receptor (EGFR)—that promote immune evasion and persistent oncogenic signaling [14]. On the other hand, lysosomes represent a highly vulnerable therapeutic node. Their acidic lumen and relatively fragile membrane render them particularly susceptible to nanomaterial accumulation, which can induce localized reactive oxygen species (ROS) generation, ionic imbalance, or osmotic stress, ultimately leading to lysosomal membrane permeabilization (LMP). The ensuing release of cathepsins into the cytosol activates irreversible cell-death pathways [15]. Furthermore, for nanotherapeutics delivering biomacromolecular cargos—including nucleic acids and proteins—efficient escape from lysosomal sequestration is a prerequisite for functional cytosolic or nuclear activity [16, 17]. Consequently, the rational design of nanomaterials that respond to lysosomal cues such as low pH, abundant proteases, or redox gradients has become a core strategy in advanced drug delivery.

In this review, we propose a conceptual shift in how the lysosome is viewed in cancer nanomedicine: rather than being regarded merely as a passive degradative endpoint, it should be recognized as a programmable therapeutic hub that can be rationally exploited for cancer therapy. Guided by this perspective, we first provide an overview of the current landscape of lysosome-centered nanomedicine and its emerging significance in cancer treatment. We then organize the field into the major functional and therapeutic modalities, including lysosome-triggered payload activation and release, endolysosomal escape for non-lysosomal intracellular targets, direct modulation of lysosomal function and signaling, and lysosome-focused therapeutic strategies driven by external stimuli or catalytic reactions. We also include discussion of receptor-guided degradation platforms such as LYTACs and structure-guided programmable systems, which provide additional opportunities to improve the specificity and versatility of lysosome-targeted interventions. Finally, we discuss the major translational challenges that currently limit the precision, selectivity, and clinical applicability of these systems, and outline future directions for the development of next-generation lysosome-centered nanotherapeutics. Through this framework, we aim to provide both an updated overview of the field and a conceptual basis for the rational design of more effective and precise lysosome-targeted cancer therapies.

## Lysosome-triggered payload activation and release

Following cellular uptake, many nanomedicines are routed to lysosomes, where the fate of the delivered payload is ultimately determined. Rather than treating lysosomal sequestration solely as a delivery barrier, an increasing number of therapeutic strategies deliberately exploit the lysosomal microenvironment as an activation site for anticancer intervention [18]. The acidic pH, high protease activity, and confined lumen of lysosomes provide reliable endogenous triggers that can initiate payload release, conversion, or function specifically within tumor cells. By engineering nanomaterials whose therapeutic activity is dormant during circulation but activated upon lysosomal entry, payload execution can be spatially restricted to the intracellular compartment most closely associated with endocytic trafficking.

### Acid-responsive drug carriers

**Fig. 1:**
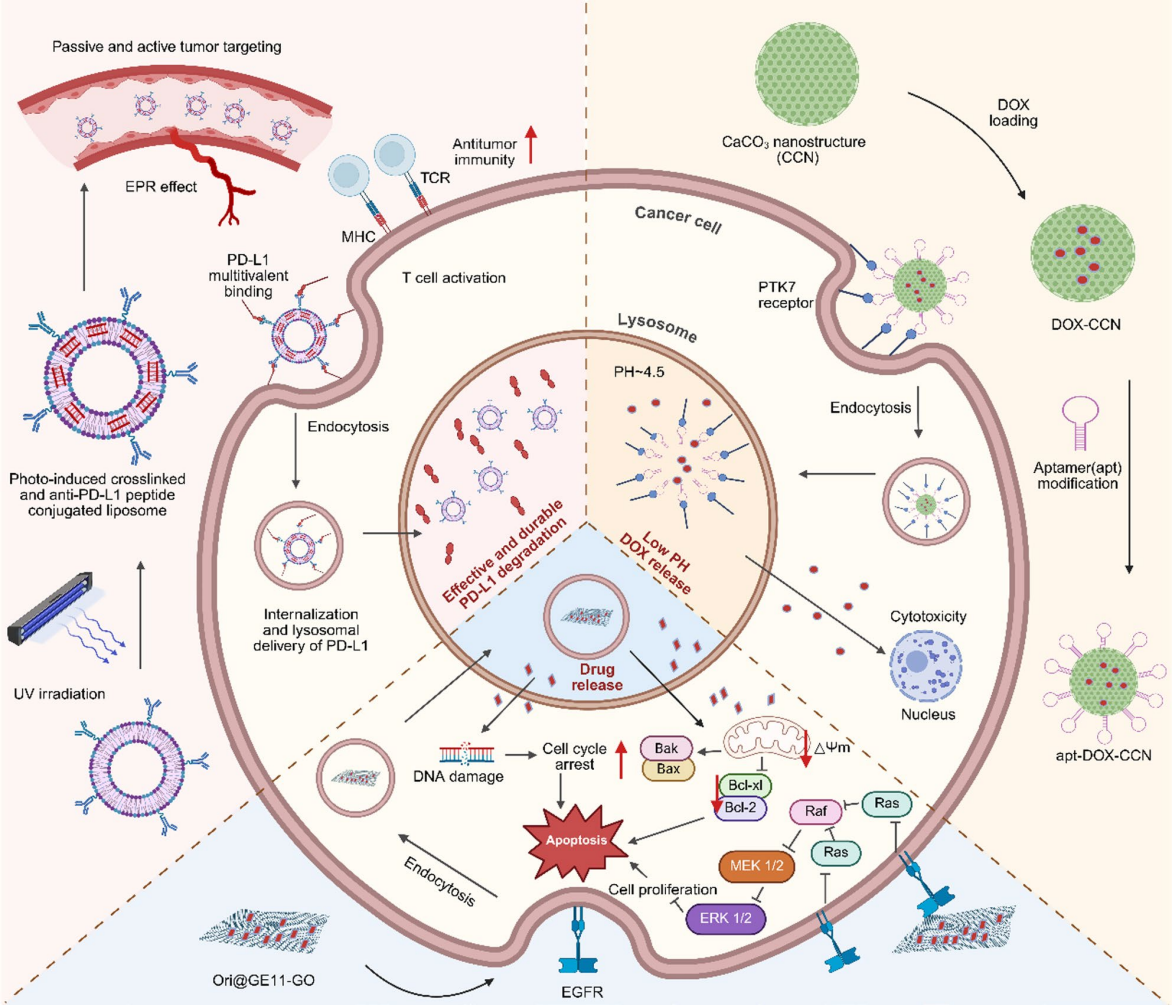
Acid-responsive nanocarriers for lysosomal delivery and activation. Lysosomal acidity serves as a universal trigger for a class of smart nanocarriers designed to release or activate their therapeutic payload specifically within cancer cells. The figure highlights three distinct platforms that exploit this mechanism: (i) Photo-crosslinked multivalent liposomes (ICB-LPs) display anti-PD-L1 peptides, which, after binding and internalization, leverage the acidic lysosomal environment to prevent PD-L1 recycling and ensure its durable degradation. (ii) Aptamer-functionalized calcium carbonate nanostructures (apt-CCNs) are designed to dissolve in the low-pH lysosome (pH 4.5–5.5), enabling burst release of encapsulated chemotherapeutics like doxorubicin directly at the target site. (iii) Peptide-targeted graphene oxide systems (Ori@GE11-GO) accumulate in lysosomes of EGFR‑overexpressing cells, where the acidic compartment facilitates drug release (oridonin), leading to mitochondrial apoptosis. By capitalizing on the conserved acidic pH of lysosomes, these nanocarriers achieve precise subcellular drug activation, enhance therapeutic specificity, and minimize off‑target effects, illustrating a convergent design principle for improving cancer therapy

Lysosomal acidity provides a universal and robust trigger for the controlled release of therapeutic payloads, with a variety of nanomaterial designs engineered to disassemble or undergo conformational changes specifically in the low-pH environment of this organelle. A prominent strategy leverages the stability and programmability of lipid-based systems. For example, photo-crosslinked liposomes displaying multivalent anti-PD-L1 peptides achieve high-avidity binding to tumor cells. Their stable architecture reroutes PD-L1 from its normal recycling pathway to lysosomal degradation, sustaining checkpoint suppression for over 72 h and restoring T-cell cytotoxicity far more durably than conventional antibodies [19]. Cancer cell membrane-coated ZnO nanoparticles dissolve in the acidic lysosome, simultaneously releasing siRNA payloads and generating zinc ions that induce osmotic pressure and rupture the organelle from within, ensuring cytosolic escape [20]. In parallel, inorganic matrices offer high drug-loading capacity. Aptamer-functionalized calcium carbonate nanostructures exemplify this approach; following receptor-mediated endocytosis, the acidic lysosomal environment dissolves the CaCO₃ core, triggering the controlled release of encapsulated doxorubicin and enabling combined therapy and imaging [21]. A pH-gated nanoadjuvant, PGN4.9, was designed to selectively target the hyper-acidic lysosomes of M2-like tumor-associated macrophages while remaining inactive in cells with less acidic lysosomes. Built from an ultra-pH-sensitive copolymer with a defined transition threshold, this system requires highly acidic lysosomal pH together with cathepsin B activity to trigger release of the TLR7/8 agonist IMDQ. Upon activation, PGN4.9 reprogrammed M2-like TAMs toward an M1-like phenotype, reduced lysosomal acidity and proteolysis, and enhanced antigen cross-presentation and CD8⁺ T cell activation. In 4T1 tumor-bearing mice, intravenous administration led to selective lysosomal accumulation in tumor-associated M2-like TAMs with minimal activation in healthy tissues. In combination with anti-PD-1, PGN4.9 induced sustained tumor regression and prolonged survival without detectable systemic toxicity [22]. Graphene oxide sheets functionalized with the targeting peptide GE11 deliver the drug oridonin selectively to EGFR-overexpressing cancer cells. Upon lysosomal accumulation, the acidic milieu facilitates high local drug release, which simultaneously induces apoptosis and suppresses oncogenic EGFR signaling pathways [23]. In summary, acid-responsive carriers represent a versatile and powerful class of lysosome-targeting nanomaterials, utilizing the organelle’s defining low-pH characteristic to achieve precise, context-dependent payload release for therapy, imaging, and pathway modulation (Fig. 1).

### Enzyme-responsive drug carriers

Lysosome-abundant enzymes, particularly cathepsins, provide a highly specific biological trigger for payload release, enabling drug activation with exceptional spatial control within the target cell. A key design employs polymeric nanoparticles sensitive to cathepsin B. One such system co-delivers paclitaxel and the AKT inhibitor capivasertib to gastric cancer cells. Upon lysosomal internalization, the overexpressed cathepsin B cleaves the prodrug matrix, triggering the simultaneous release of both agents. This coordinated action suppresses the PI3K-AKT survival pathway while inducing mitotic arrest, generating a potent synergistic antitumor effect with reduced systemic toxicity [24]. To further enhance tumor selectivity, dual enzyme–responsive systems have been engineered. A sophisticated nanovector for pancreatic cancer treatment incorporates a matrix metalloproteinase-9 (MMP-9)–cleavable polyethylene glycol (PEG) shield and a cathepsin B–sensitive linker tethering gemcitabine to a quantum dot core. In the TME, MMP-9 first cleaves the PEG corona to expose targeting ligands, enhancing cellular uptake. Following endocytosis, lysosomal cathepsin B then cleaves the linker to release the active gemcitabine metabolite, protecting it from deactivation and significantly boosting its intracellular concentration and efficacy [25]. Collectively, enzyme-responsive carriers exemplify a biomimetic and precise release strategy, leveraging the unique protease activity within the lysosome to achieve tumor-selective drug activation and enhanced therapeutic outcomes.

## Lysosome escape

In many cancer therapies, the intended molecular targets—including oncogenic kinases, transcriptional regulators, and DNA—are located in the cytosol or nucleus rather than within the endolysosomal compartment. Following cellular uptake, however, nanomedicines are frequently sequestered in endosomes and lysosomes, where cargo degradation or entrapment can severely limit therapeutic efficacy. Consequently, efficient escape from the endolysosomal pathway represents a critical prerequisite for enabling these agents to reach their intracellular targets and exert antitumor activity [26]. To address this barrier, a diverse array of nanomaterial-based strategies has been developed to promote lysosomal membrane destabilization and facilitate cytosolic delivery.

### pH-responsive type

Distinct from acid-responsive drug carriers where payload release occurs without lysosomal membrane disruption, the following strategies aim to physically compromise the lysosomal membrane to enable cytosolic escape of nanocarriers. The acidic pH of lysosomes provides a universal trigger for nanocarriers designed to disrupt the organelle’s integrity and facilitate escape. A primary strategy involves the use of charge-switchable materials. For instance, smart nanomicelles are engineered to convert from a negative to a positive surface charge upon encountering the acidic TME and endolysosomal compartments. This charge reversal enhances cellular membrane association and uptake. Once inside acidic lysosomes, the protonatable groups within the micelle core induce a “proton-sponge” effect, causing osmotic swelling and subsequent vesicle rupture that enables the efficient cytosolic deposition of co-delivered chemotherapeutics like doxorubicin and paclitaxel [27]. Similarly, modular albumin-based nanocarriers utilize a polyhistidine core. Following tumor-targeted delivery and proteolytic activation in the TME, the exposed cationic micelle is internalized. Within the acidic lysosome, the imidazole groups of histidine residues become protonated, which both buffers the organelle’s pH and induces membrane destabilization, thereby driving the escape and cytosolic release of encapsulated paclitaxel [28]. Beyond polymeric systems, ionizable lipid-based nanoparticles represent another powerful pH-responsive platform for endosomal escape. These lipids remain neutral or minimally charged at physiological pH (7.4), ensuring safe systemic circulation with reduced cytotoxicity. Upon entry into acidic endosomes (pH 6.5–5.4), the amino head groups of nonlamellar ionizable lipids undergo protonation, acquiring amphiphilic character and cationic charge density. The resulting electrostatic interaction with the negatively charged endosomal membrane, combined with the large wedge-shaped lipid tail structure, induces localized membrane disruption and thinning. This pH-sensitive amphiphilic membrane destabilization enables efficient endosomal escape and cytosolic release of nucleic acid cargoes such as siRNA and mRNA, offering a structurally tunable mechanism for enhanced intracellular delivery [29]. Collectively, pH-responsive systems exploit the endolysosomal acidification cascade to trigger morphological or charge transitions that physically compromise the lysosomal membrane, serving as a foundational and widely applicable escape mechanism.

### Redox-responsive type

The high concentration of reducing agents like glutathione (GSH) within the cytosol, contrasted with the oxidizing environment of lysosomes, offers a biochemical gradient for designing escape mechanisms. Nanocarriers incorporating disulfide bonds remain stable during circulation and lysosomal transit but undergo reductive cleavage upon cytosolic entry or lysosomal rupture, facilitating drug release and carrier disassembly. One approach uses folate-targeted PLGA nanoparticles crosslinked with disulfide bonds. After receptor-mediated endocytosis, the reducing cytosolic environment triggers the degradation of the nanocarrier, allowing it to bypass lysosomal sequestration and achieve on-demand paclitaxel release directly in the cytosol [30]. A more integrated “lysosomal bomb” design combines pH and redox responsiveness. A platform based on vaterite calcium carbonate and disulfide-crosslinked alginate encapsulates doxorubicin. The acidic lysosome triggers CaCO₃ expansion to physically rupture the membrane, while intracellular GSH cleaves the disulfide bonds to ensure selective drug release in tumor cells [31]. Further expanding the scope, a hypoxia-responsive polypeptide nanocarrier is engineered to self-assemble into core–shell micelles that encapsulate cytochrome c and selectively release it within oxygen-deprived tumor tissue. The micelles remain stable under normoxia yet trigger bond cleavage and cargo liberation in hypoxic environments, while their amphiphilic architecture enables efficient cellular uptake and rapid endo-lysosomal escape. In HepG2 cells, this lysosome-bypassing delivery markedly enhances cytochrome c-driven cytotoxicity under hypoxia, highlighting a biodegradable polypeptide platform capable of exploiting TME cues for targeted intracellular protein therapy [32]. Additionally, tellurium-based nanoconstructs release components in response to GSH that elevate lysosomal pH and osmotic pressure, functionally disrupting the organelle to block protective autophagy and enhance phototherapy [33]. In summary, redox-responsive strategies provide a highly specific trigger for lysosomal disruption or carrier degradation, often working in concert with other microenvironmental cues to enable precise cytosolic delivery.

### Enzyme-responsive type

In contrast to enzyme-responsive drug carriers (see above) where enzymatic cleavage releases small-molecule drugs from carrier matrices, the approaches in this subsection employ enzymatic reactions to directly destabilize the lysosomal membrane, facilitating escape of the carrier and its macromolecular cargo. Lysosome-abundant enzymes, such as cathepsins and lipases, can be harnessed to catalyze the activation of membrane-disruptive functionalities. A biomimetic approach involves nanoparticles cloaked in a functional cell membrane. One such design for glioblastoma therapy incorporates a lipid core that, upon lysosomal uptake, undergoes enzyme-mediated peroxidation. This chemical transformation destabilizes the lysosomal membrane from within, facilitating escape and the release of temozolomide into the cytosol [34]. A more complex nanoimmunomodulator employs a dual-responsive structure. Its outer shell is cleaved by enzymes in the TME, unmasking a cationic core. Following endocytosis, this cationic surface promotes lysosomal membrane disruption, enabling the simultaneous cytosolic delivery of siRNA and the endoplasmic reticulum-specific localization of a photosensitizer for combined immunotherapy [35]. Similarly, a “nano-domino” construct is activated by intracellular enzymes to shed an inert shell. This activation initiates a cascade where the platform escapes lysosomes and releases a CRISPR/Cas9 system to disable the anti-apoptotic protein Bcl-2, thereby reprogramming cancer cells to be more susceptible to photodynamic therapy (PDT) [36]. These examples illustrate how enzyme-responsive designs can confer a high degree of spatial and biochemical specificity, triggering escape mechanisms only in the desired cellular context.

### Material-enabled lysosomal escape

**Fig. 2:**
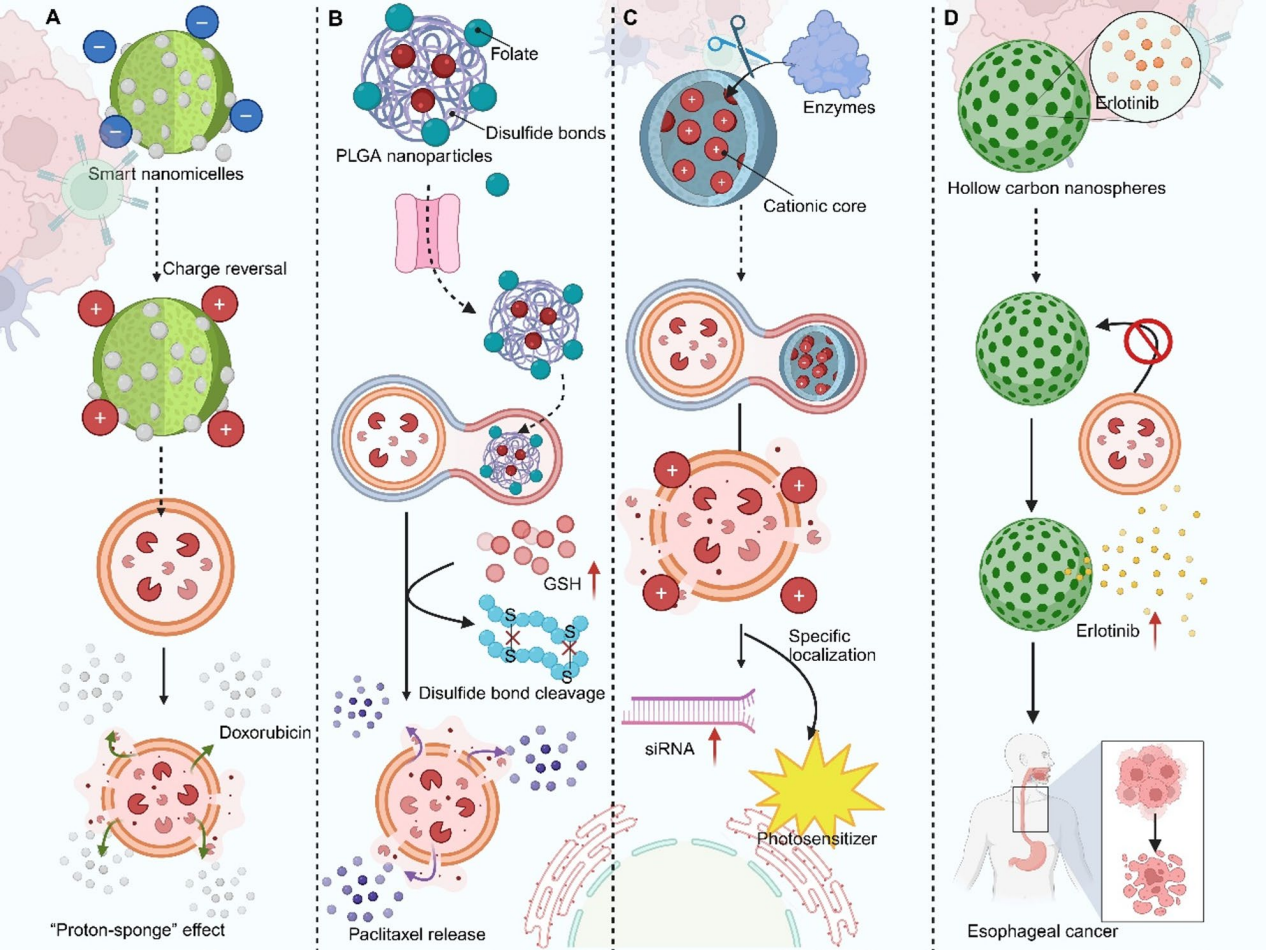
Mechanistic diagrams of four major lysosomal escape strategies. Lysosomal escape strategies are engineered to overcome the endolysosomal barrier, thereby ensuring therapeutic cargos reach the cytosolic compartment where they exert their activity. The figure contrasts five nanomaterial-based approaches: (**A**) pH-responsive escape utilizes the proton-sponge effect of charge-switchable nanomicelles to rupture endolysosomes; (**B**) Redox-responsive escape employs disulfide-crosslinked nanoparticles that degrade in the high-GSH cytosol after internalization; (**C**) Enzyme/dual-stimuli-responsive escape relies on TME-specific enzyme cleavage and polymer self-escape for triggered payload release; (**D**) Material-directed bypass uses porous carbon nanospheres that evade lysosomal capture, enabling direct cytoplasmic access. These strategies collectively enhance the bioavailability and efficacy of therapeutics by bypassing lysosomal degradation

Beyond the pH- and enzyme-responsive mechanisms discussed above, certain nanomaterials possess intrinsic physicochemical properties that enable lysosomal escape through distinct pathways, such as morphology-dependent trafficking, gas generation, or ion-mediated membrane disruption. Certain inorganic nanomaterials possess intrinsic physicochemical properties that promote lysosomal escape through direct physical interaction or dissolution. Carbon-based structures, such as hollow carbon nanospheres, leverage their ultra-small porosity and spherical morphology to facilitate rapid cellular uptake. Their chemically inert nature and specific size allow them to evade prolonged lysosomal trapping, enabling direct cytoplasmic access and sustained drug release, as demonstrated with erlotinib delivery for esophageal cancer [37]. A DNAzyme capsule with a Mn²⁺/Zn²⁺–phytate shell disassembles in the acidic lysosome, releasing its gene-editing cargo. The co-released metal ions and polymeric transfection agents (e.g., polyethyleneimine) then work synergistically to buffer the organelle and disrupt its membrane [38]. Furthermore, sophisticated nanoformulations can be designed to generate gases (e.g., CO₂) upon lysosomal degradation. The rapid expansion of these gases creates high internal pressure, physically rupturing the lysosome to ensure the cytosolic release of drugs that reverse ferroptosis resistance in triple-negative breast cancer [39]. Thus, inorganic and hybrid nanomaterials provide a diverse toolkit for lysosomal escape, leveraging dissolution, phase transitions, gas generation, or membrane fusion to achieve cytosolic delivery (Fig. 2).

## Lysosome function modulation

Strategic modulation of lysosomal function has emerged as a distinct therapeutic dimension in cancer nanomedicine. Rather than aiming solely at lysosomal disruption or cargo release, these approaches deliberately tune key lysosomal properties—including luminal pH, enzymatic activity, membrane stability, and autophagic flux—to reprogram cancer cell survival and stress-adaptation pathways. By reshaping lysosome-dependent metabolic recycling, drug sequestration, and signaling outputs, such interventions can sensitize tumors to therapy, overcome resistance, and, in certain contexts, directly trigger selective cell death.

### Lysosomal pH modulation

The acidic pH of lysosomes is essential for the activity of hydrolytic enzymes and autophagic degradation, making its perturbation a potent strategy to cripple these pro-tumorigenic processes. One approach employs nanocatalysts that consume intralysosomal protons. For example, a tumor-targeting heterojunction nanocatalyst, upon ultrasonic activation, not only generates hydrogen gas to suppress mitochondrial function but also degrades to release phosphate anions that consume protons, thereby raising lysosomal pH, inactivating acid hydrolases, and blocking protective autophagy [40]. Similarly, ultra-pH-sensitive micelles (UPSM) are engineered with an exceptionally sharp pH-responsive transition. Upon accumulating in lysosomes of KRAS-mutant pancreatic cancer cells, they undergo a micelle-to-unimer shift that effectively buffers the organelle’s acidity. This suppresses lysosomal protein catabolism—a key nutrient source for these tumors—and synergistically enhances the cytotoxicity of co-delivered chemotherapeutic agents [41]. Beyond chemical buffering, nanomaterials can directly target the molecular machinery regulating pH. A black phosphorus-based nanoplatform is designed to silence the ClC-3 chloride channel, a key regulator of lysosomal acidification. By inhibiting ClC-3, the platform prevents the acidification process, which concurrently inhibits autophagy and reverses the lysosomal sequestration of chemotherapeutic drugs like doxorubicin, thereby overcoming a major resistance mechanism [42]. Additionally, polymeric micelles can achieve pH modulation through intrinsic chemical properties. pH-sensitive micelles loaded with epirubicin specifically localize to lysosomes and induce dose-dependent alkalization, which impairs autophagic flux and sensitizes cancer cells to the drug’s cytotoxic effects [43]. Expanding on this theme, peptide-coated DNA nanostructures (DNs) have been shown to modulate lysosomal pH in a surface-coating-dependent manner. Using a 6‑helix bundle scaffold, coating with an aurein‑containing peptide (EE DNs) induced sustained lysosomal alkalization at low concentrations, which triggered stimulator of interferon genes (STING) upregulation and immune signaling without membrane rupture. Conversely, high concentrations of the same EE DNs caused lysosomal alkalinization that progressed to vesicle rupture and mitochondrial damage, driving potent cytotoxicity. This work demonstrates that rational peptide functionalization can enable both mild pH elevation for immunomodulation and severe alkalization-induced lysosomal disruption for cancer cell killing [44]. Collectively, these strategies highlight lysosomal pH as a central therapeutic node, where its disruption can simultaneously inhibit autophagy, impair nutrient recycling, and enhance the efficacy of co-administered therapies.

### Autophagic flux modulation

Autophagy is a multi-step process culminating in lysosomal degradation; inhibiting the fusion of autophagosomes with lysosomes (autophagic flux) leads to the toxic accumulation of cellular debris and induces stress. Nanomaterials can physically interfere with this fusion process. Nanostructures like NBP/TiO₂ have been shown to localize within cells and block autophagosome-lysosome fusion, leading to a buildup of undegraded autophagosomes. This inhibition, especially when combined with proteasome inhibitors, synergistically amplifies cellular stress and cell death [45]. Alternatively, platforms can exploit and hijack the autophagic pathway for drug delivery. Polydopamine-coated mesoporous silica nanoparticles are internalized and trafficked to lysosomes via autophagy-related pathways. By understanding and potentially inhibiting the subsequent exocytic routes (mediated by Rab proteins) that remove these nanoparticles, their therapeutic retention and efficacy can be significantly enhanced [46]. Thus, modulating autophagic flux provides a dual benefit: it can directly induce cytotoxic stress by halting a critical clearance pathway, and it can be leveraged to improve the intracellular fate and performance of therapeutic nanomaterials.

### Lysosomal membrane integrity disruption

Directly compromising the lysosomal membrane, a process known as LMP, leads to the catastrophic release of potent hydrolases into the cytosol, triggering various forms of cell death. This can be achieved through chemically induced mechanical stress. For instance, a peptide precursor can be designed to undergo enzyme-triggered internalization and light-activated self-assembly within lysosomes. The resulting formation of rigid, interlocking nanofibrils and nanorods generates physical force sufficient to rupture the organelle from within [47]. Similarly, supramolecular transformers like small-molecule conjugates can self-assemble into nanoparticles for delivery, then morph into rigid fibrils inside the acidic lysosome, mechanically disrupting its function and inducing cell death, an effect further amplified by light [48]. Physical force from external fields offers another direct mechanism. Magnetically responsive nanoparticles can be engineered to self-assemble into large intracellular aggregates under a rotating magnetic field. These aggregates exert substantial mechanical force, directly breaching the lysosomal membrane. Magnetically responsive nanoparticles engineered for EGF-mediated uptake into cancer cells are shown to self-assemble into elongated intracellular aggregates capable of exerting substantially amplified mechanical forces. Zinc-doped iron oxide cores provide high magnetization, enabling low-frequency rotating fields to drive aggregate formation that breaches both plasma and lysosomal membranes. The resulting lysosomal rupture and release of hydrolases into the cytosol initiate programmed cell death and necrosis. This mechanically driven disruption of lysosomal integrity highlights a powerful lysosome-targeting nanomechanical strategy for cancer eradication [49]. A parallel magneto-mechanical strategy uses ultra-small superparamagnetic nanoparticles that, when activated by a low-frequency field, generate sufficient force within lysosomes to cause permeabilization and cathepsin release, effectively killing supportive cells in the TME [50]. Acoustic energy can also be harnessed to inflict mechanical damage, as seen with hybrid nanomachines that localize to lysosomes and, upon ultrasound activation, generate intense local stress to rupture the membrane [51]. Furthermore, chemically generated pressure provides a potent disruptive force. A lipid-droplet-derived nanoconstruct, activated by ultrasound and Fenton chemistry, produces ROS that permeabilize the lysosomal membrane, releasing sequestered drugs and restoring chemosensitivity in resistant cells [52].

### Lysosomal enzyme activity modulation

Beyond gross membrane disruption, a more nuanced strategy involves interfering with the activity of lysosomal enzymes to alter cellular metabolism and drug handling. A prominent example focuses on overcoming lysosomal drug sequestration, a common resistance mechanism. A self-assembling prodrug platform based on hydroxycamptothecine–silane conjugates is designed to traffic to lysosomes. The acidic environment triggers a dual response: it initiates the release of components that destabilize the lysosomal membrane, while simultaneously inducing a condensation reaction that transforms the nanoparticles into larger silicon assemblies outside the lysosome. This ingenious process not only disrupts the organelle but also actively prevents the drug from being trapped and inactivated within it, thereby dramatically enhancing its potency against resistant cancer models [53]. This approach illustrates that modulating the lysosomal environment and its interaction with therapeutics can be a highly effective strategy to reverse drug resistance and restore efficacy.

### Signaling pathway intervention

**Fig. 3:**
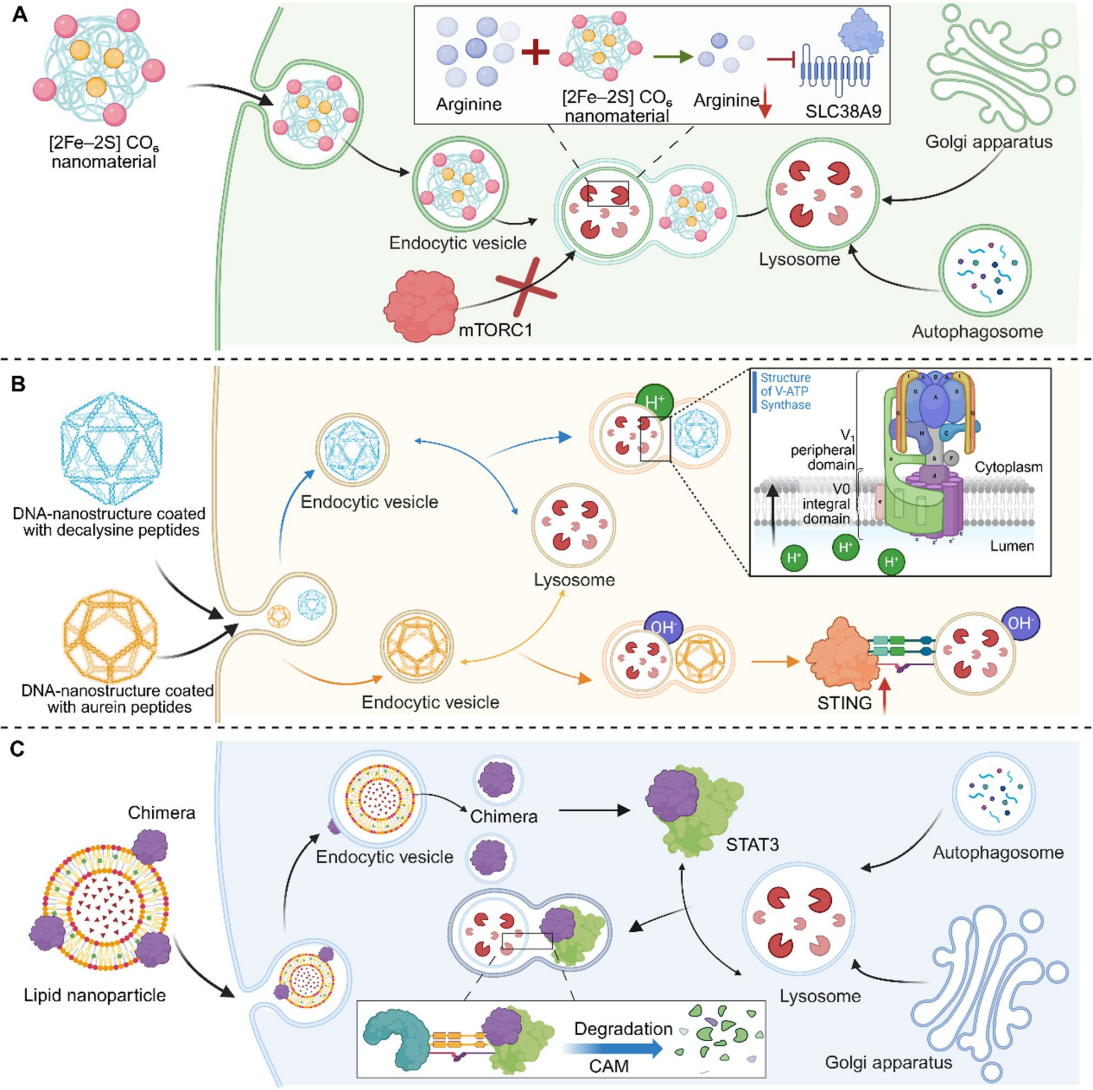
Targeting lysosome-associated signaling pathways for cancer therapy. The lysosome functions as a critical signaling hub, which can be therapeutically modulated by nanomaterials to disrupt key oncogenic pathways. This figure illustrates three distinct targeting strategies: (**A**) Nanoscale inhibitors (e.g., [2Fe–2 S]CO₆) deplete intralysosomal arginine to block SLC38A9 sensing and mTORC1 recruitment, suppressing tumor growth. (**B**) Programmable DNA nanostructures coated with specific peptides (decalysine or aurein) precisely regulate lysosomal pH—promoting acidification for metabolic remodeling or alkalization for STING pathway activation. (**C**) Lipid nanoparticles deliver chimeric proteins that hijack the chaperone-mediated autophagy (CMA) pathway to recruit and degrade oncogenic transcription factors like STAT3. Collectively, these approaches demonstrate how engineered nanomaterials can precisely intercept, modulate, or exploit lysosomal signaling nodes to achieve targeted anticancer effects

Beyond directly altering lysosomal physiology, an advanced therapeutic strategy involves targeting the lysosome as a central signaling hub to intercept critical pro-tumorigenic pathways. This approach leverages nanomaterials to precisely modulate the molecular machinery associated with the lysosomal surface or its lumen, thereby reprogramming cellular metabolism and fate. A key focus is disrupting nutrient-sensing pathways, exemplified by a lysosome-directed nanoinhibitor designed to degrade intralysosomal arginine. This platform, based on a [2Fe–2 S]CO₆ construct, prevents the activation of the arginine sensor SLC38A9, which in turn blocks the recruitment and activation of the master growth regulator mechanistic target of rapamycin complex 1 (mTORC1) at the lysosomal membrane. This mechanism achieves ultra-potent and highly selective mTORC1 inhibition, exhibiting a stark differential effect between normal and bladder cancer cells. When combined with PDT, it effectively suppresses tumor regrowth in vivo, demonstrating how lysosome-targeted metabolic interception can enact precise pathway blockade [54]. Furthermore, the lysosome’s degradative capacity can be co-opted for targeted signal disruption via specialized autophagy pathways. Lipid nanoparticles have been engineered to deliver chimeras that hijack Chaperone-mediated autophagy (CMA). These chimeras bind the oncogenic transcription factor signal transducer and activator of transcription 3 (STAT3) and recruit it to the lysosome for degradation [55]. The strategy is enhanced by coupling it with a fasting-mimicking diet, which upregulates the CMA machinery and the targeted receptor on tumor cells, creating a metabolically primed environment for efficient oncoprotein clearance and tumor suppression (Table 1) (Fig. 3).

**Table 1:** Summary of nanomaterial strategies for modulating lysosomal function in cancer therapy

Modulation type	Primary target	Representative nanomaterials/designs	Core mechanism	Effect on cancer	Ref
Lysosomal pH modulation	Lysosomal acidification (protons, ClC-3 channel)	Tumor-targeting, heterojunction nanocatalysts	↑ Hydrogen gas generation, ↓ protons, ↑ lysosomal pH	↓Acid hydrolases, ↓autophagy, ↑chemotherapeutic drug efficacy	[40]
		Ultra-pH-sensitive micelles (UPSM)	Buffering lysosomal acidity via micelle-to-unimer transition @Micelle transition→ lysosomal alkalization → blocked autophagic flux	↓Lysosomal protein catabolism	[41]
		Black phosphorus-based nanoplatform	Silence ClC-3 channel→ ↓Acidification, ↓Autophagy, Reversing lysosomal drug sequestration	↓Resistance mechanism, ↑drug action	[42]
		PH-sensitive polymeric nanoparticles	Inducing dose-dependent lysosomal alkalization@↑Lysosomal pH	↓Autophagic flux, ↑cytotoxic effects	[43]
Autophagic flux modulation	Autophagosome-lysosome fusion; rab-mediated exocytosis	NBP/TiO₂ nanostructures	↓Fusion of autophagosomes and lysosomes → ↓autophagic flux	↑Cellular stress, ↑cell death	[45]
		polydopamine-coated mesoporous silica nanoparticles	↓Autophagic flux→↓lysosomal function@↓rab protein-mediated exocytic routes, ↑nanoparticle retention in lysosomes	↑Sensitivity to chemotherapeutic agents, ↑drug efficacy	[46]
Lysosomal membrane integrity disruption	Lysosomal membrane (lipid bilayer)	Light-activated peptide precursors, small-molecule conjugates	Enzyme-triggered internalization → light-activated self-assembly → lysosomal membrane disruption	Rupturing the membrane, ↑cell death	[48]
		Magnetic nanoparticles	Nanofibril formation or magnetic aggregation →physical force	Rupturing the membrane	[49]
		Ultra-small superparamagnetic nanoparticles	↑Permeabilization, ↑cathepsin release	↓Supportive cells	[50]
		Acoustic energy	↑Mechanical damage,↑ intense local stress	Rupturing the membrane	[51]
		Lipid-droplet-derived nanoconstructs	↑Lipid peroxidation, ↑ROS production	Permeabilizing the membrane, releasing sequestered drugs, Restoring chemosensitivity in resistant cells	[52]
Lysosomal enzyme activity modulation	Lysosomal hydrolytic enzymes; drug sequestration	Hydroxycamptothecine–silane conjugate self-assembling prodrug platform	Releasing components → ↓the lysosomal membrane, ↑condensation reaction, ↓drug trapping	Overcoming lysosomal drug sequestration, ↑potency against resistant cancer models, ↓drug resistance	[53]
Signaling pathway intervention	Lysosome-associated signaling hubs (mTORC1, STING, CMA machinery)	[2Fe–2 S]CO₆-based nanoinhibitor	↓Intralysosomal arginine, ↓SLC38A9-mTORC1 signaling	Achieving ultra-potent and highly selective mTORC1 inhibition	[54]
		programmable DNA-nanostructures	Modulating lysosomal pH →trigger metabolic reprogramming or STING pathway activation	Lysosomal swelling or rupture	[44]
		CMA-hijacking lipid nanoparticles	Hijacking CMA, ↓oncogenic transcription factor STAT3	↑Oncoprotein clearance and tumor suppression.	[55]

## Lysosome-directed protein degradation: LYTACs and beyond

LYTACs are a class of bifunctional degraders designed to eliminate extracellular and membrane-associated proteins by redirecting them to the lysosomal pathway. In contrast to proteasome-based degraders such as proteolysis-targeting chimera (PROTAC), LYTACs operate through endocytic or vesicular trafficking mechanisms, typically by simultaneously engaging a target protein and a lysosome-trafficking receptor to induce internalization and lysosomal degradation. This conceptual framework enables the pharmacological removal of disease-associated surface proteins that are inaccessible to intracellular degradation systems [56]. Building on this core principle, LYTAC technologies have rapidly diversified in both receptor usage and molecular design, giving rise to a broad spectrum of strategies tailored for tumor-selective targeting, enhanced degradation efficiency, and improved therapeutic precision in cancer [57].

### Receptor-guided LYTAC strategies

#### ASGPR-targeted LYTACs

Asialoglycoprotein receptor (ASGPR) is an endocytic lectin receptor that binds terminal N-acetylgalactosamine (GalNAc) with exceptionally high affinity and mediates rapid internalization of its ligands. Upon engagement, the receptor–cargo complex is routed through early and late endosomes toward lysosomal degradation, providing a highly efficient pathway for clearing extracellular and membrane-associated proteins [58, 59]. For instance, GalNAc-engineered amphiphilic peptides self-assemble into nanospheres that function as modular LYTACs, binding ASGPR to drive lysosomal trafficking of membrane and extracellular proteins. Conjugation with anti-CD24 enables selective depletion of the CD24–Siglec-10 axis, restoring macrophage phagocytosis through enforced lysosomal degradation of the GPI-anchored ligand. Coupling the nanospheres with glucose oxidase further amplifies macrophage activation and suppresses tumor growth in vivo [60]. In addition, polymer-based LYTACs couple PD-L1–binding motifs with ASGPR ligands to drive liver tumor–specific lysosomal degradation of PD-L1 while sparing its expression on host immune cells. Through ASGPR-mediated endocytosis, this nanoplatform selectively depletes PD-L1 in hepatocellular carcinoma cells without affecting PD-L1 levels in dendritic cells or macrophages. Integration with whole-body PD-L1 PET imaging reveals that host-cell, rather than tumor-cell, PD-L1 predominates in determining in vivo responsiveness to anti-PD-1 therapy [61]. Similarly, tri-GalNAc–conjugated degraders are engineered to exploit the liver-specific ASGPR as a lysosomal shuttle, enabling cell-type-restricted clearance of extracellular and membrane proteins. By linking peptide or antibody binders to a tri-GalNAc motif, these GalNAc-LYTAC nanomaterials drive efficient ASGPR-mediated endocytosis and lysosomal trafficking. EGFR-directed GalNAc-LYTACs suppress downstream signaling more effectively than antibody inhibition, while a 3.4-kDa peptide–GalNAc construct degrades integrins and curtails cancer cell proliferation. Site-specific tri-GalNAc conjugation on antibody scaffolds further improves pharmacokinetics in vivo [62].

#### IGF2R-directed LYTACs

The insulin-like growth factor 2 receptor (IGF2R) serves as a versatile lysosomal trafficking receptor for targeted protein degradation. Nanomaterials engineered to engage IGF2R leverage its endocytic function to shuttle specific membrane proteins to lysosomes, offering a potent strategy for cancer therapy [63]. For example, programmable DNA-assembled LYTAC nanostructures have been designed to present multivalent aptamers that simultaneously bind IGF2R and disease-associated targets like PD-L1 or vascular endothelial growth factor receptor 2 (VEGFR2). This tunable, multivalent architecture markedly enhances binding avidity and internalization, driving efficient lysosomal degradation. Consequently, depletion of PD-L1 restores T-cell activity while loss of VEGFR2 suppresses tumor growth, demonstrating dual immunomodulatory and antitumor effects. Furthermore, the modularity of DNA-based assembly allows for precise spatial organization, overcoming affinity limitations and expanding the efficacy of lysosome-directed clearance [64]. In parallel, small molecule-based degraders have been developed by conjugating the EGFR inhibitor erlotinib to an IGF2R-binding aptamer. This construct, termed LY-dE#5, selectively recruits diverse EGFR variants—including wild-type and drug-resistant mutants such as L858R/T790M and C797S—to the lysosomal pathway. By enforcing lysosomal clearance rather than kinase inhibition, LY-dE#5 potently suppresses EGFR-driven tumor growth and surpasses the efficacy of current tyrosine kinase inhibitors like Osimertinib, highlighting a promising route to overcome therapeutic resistance [65]. In addition to nucleic acid–based and small-molecule conjugates, IGF2R-targeted liposomal nanoplatforms have been engineered to enable lysosome-specific drug delivery. An IGF2R-targeted liposomal nanoplatform has been engineered to deliver hydroxychloroquine (HCQ) with lysosomal precision. This system exploits metabolic vulnerabilities in tumors: a fasting-mimicking diet amplifies autophagy and upregulates IGF2R on malignant cells, enhancing tumor-specific accumulation of the liposomes. By concentrating HCQ within autophagic lysosomes, the platform disrupts survival pathways and sensitizes tumors to autophagy blockade, yielding marked suppression of tumor growth with minimal systemic toxicity [66]. In parallel, protein engineering has yielded a next-generation platform termed sLYTACs, which employ engineered IGF2 mutants fused to targeting antibodies. These chimeras bind IGF2R with high affinity and selectivity, avoiding proliferative signaling through IGF1R. sLYTACs enable efficient internalization and lysosomal clearance of oncogenic membrane proteins, including tyrosine kinase inhibitor -resistant EGFR mutants and human epidermal growth factor receptor 2 (HER2). By enforcing coordinated degradation across multiple resistance nodes, sLYTACs potently suppress the growth of drug-resistant tumors in vivo [67]. Collectively, IGF2R-directed LYTACs exemplify a versatile and potent nanomaterial strategy for achieving targeted protein degradation, with applications spanning nucleic acid nanostructures, small molecule conjugates, liposomal delivery, and engineered proteins to overcome cancer resistance mechanisms.

#### Tumor-specific receptor-targeted LYTAC

To enhance tumor selectivity and reduce off-target effects, recent LYTAC platforms have been engineered to hijack receptors that are specifically enriched or overexpressed on cancer cells. This approach confines lysosomal degradation to the TME, improving therapeutic precision. For instance, the folate receptor α (FRα) has been identified as a previously unrecognized lysosome-trafficking receptor. Polyvalent FRTAC nanodegraders coupling FRα-binding modules to anti-EGFR or anti-PD-L1 ligands efficiently crosslink targets for lysosomal internalization. FR-Ctx depletes EGFR to suppress tumor proliferation, while FR-Atz degrades PD-L1, reshaping the TME into an immunostimulatory state and enhancing T-cell cytotoxicity in vivo [68]. Similarly, glypican-3 (GPC3), a proteoglycan overexpressed in liver cancers, has been harnessed to create GLTACs. By linking a GPC3-binding peptide to ligands for targets like PD-L1 or c-Met, GLTACs assemble ternary complexes that undergo rapid, tumor-specific internalization and lysosomal routing. Notably, the PD-L1–directed GLTAC WP0 triggers robust T-cell–mediated cytotoxicity exclusively in GPC3⁺ tumor cells [69]. In addition to FRα and GPC3, the HER2 receptor has been repurposed as a tumor-selective lysosomal shuttle. A HER2-binding peptide was converted into a programmable HerTAC nanodegrader that tethers PD-L1 to HER2, driving high-efficiency lysosomal degradation of PD-L1 in HER2⁺ cells. This optimized stapled HerTAC exhibits improved stability and pharmacokinetics, exerting potent antitumor activity with low systemic toxicity. The same design principle has been extended to degrade other challenging targets like VISTA and MIF, underscoring the platform’s modularity [70]. Furthermore, sortilin, a tumor-enriched sorting receptor, has been leveraged to develop mRNA-encoded lysosomal targeting chimera (MedTAC) strategy. Guided by computational design with AlphaFold-Multimer, a non-endogenous sortilin binder enables modular constructs that rapidly route oncogenic proteins like PTK7 to lysosomes. In breast cancer models, a single low dose of MedTAC (PTK7) induces profound and sustained protein degradation, leading to marked tumor control and prolonged survival without systemic toxicity [71]. More recently, a supramolecular approach has been devised using self-assembling amphiphilic peptides that form nanofibers integrating carbonic anhydrase IX (CAIX) recognition. These Supra-LYTACs co-assemble with target proteins to form dynamic ternary complexes that are selectively internalized into CAIX-expressing tumor cells, where multivalent engagement accelerates lysosomal degradation of the captured protein [72]. In summary, tumor-specific receptor-targeted LYTACs—including those engaging FRα, GPC3, HER2, sortilin, and CAIX—provide a powerful and expanding set of nanomaterial platforms that confine lysosomal protein degradation to malignant tissue, thereby enhancing specificity and therapeutic index in cancer therapy.

#### Non-classical lysosome-trafficking receptors for LYTACs

**Fig. 4:**
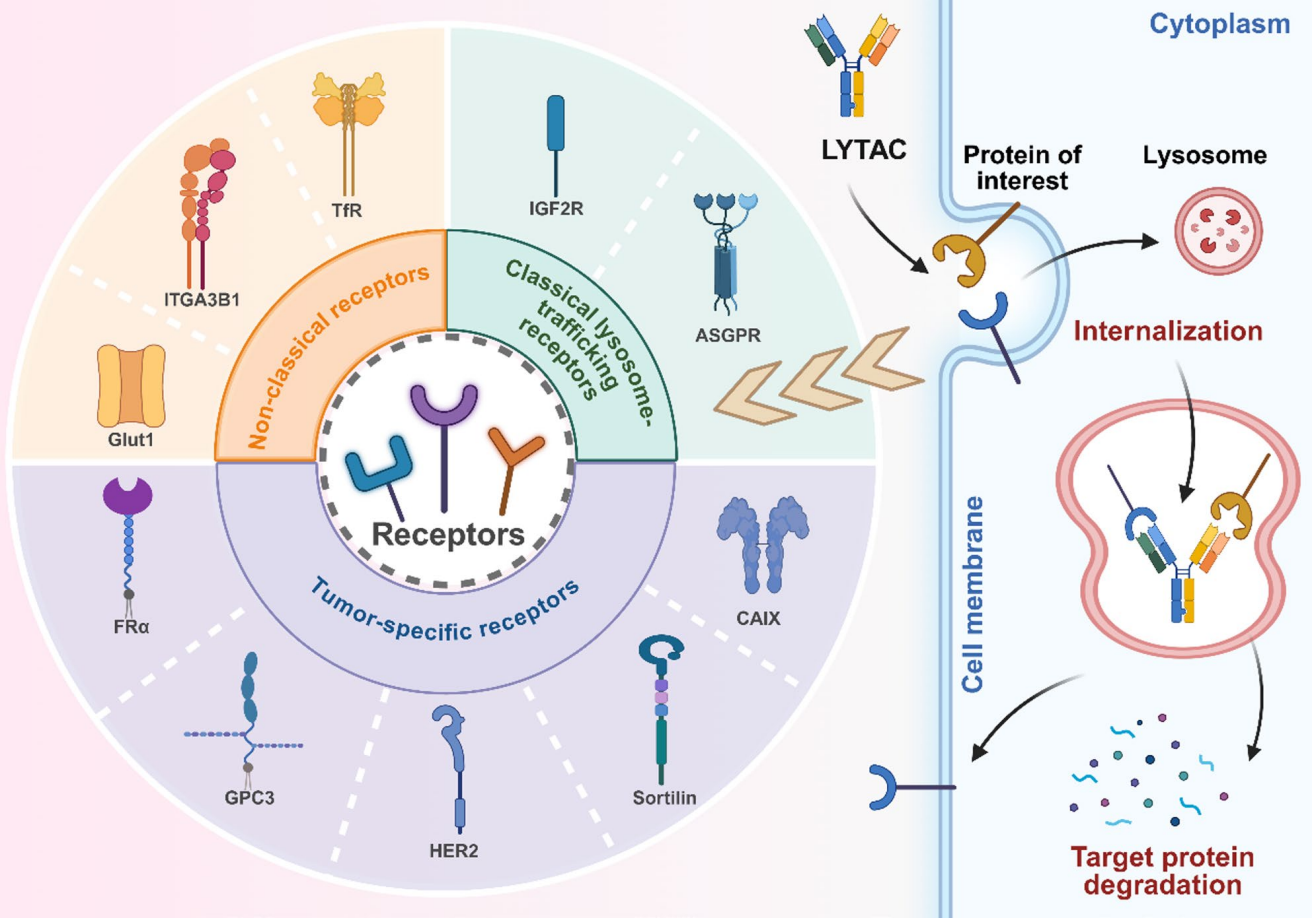
Schematic illustration of common receptor-guided LYTAC strategies. LYTAC platforms are systematically categorized according to three major classes of lysosome-trafficking receptors: tumor-targeting receptors—including folate receptor alpha (FRα), glypican-3 (GPC3), human epidermal growth factor receptor 2 (HER2), and sortilin—which are often overexpressed in malignant cells and enable cancer-selective degradation; classical endocytic receptors—such as the asialoglycoprotein receptor (ASGPR) and the insulin-like growth factor 2 receptor (IGF2R)—which are widely utilized for efficient, broad-spectrum internalization and lysosomal routing; and non-classical endocytic receptors—for example, certain integrins and lectins—that can be co-opted for induced internalization under specific pathological conditions. Each LYTAC is engineered as a bifunctional molecule that simultaneously engages one of these receptors and a target protein of interest (e.g., PD-L1, EGFR, or CD24). Upon binding, the complex undergoes receptor-mediated endocytosis, traffics through early and late endosomal compartments, and is ultimately delivered to the lysosome, where the target protein is proteolytically degraded. This modular, receptor-categorized approach allows for tunable specificity—ranging from tumor-restricted to tissue-wide or context-induced protein clearance—offering a versatile and programmable strategy to overcome drug resistance, modulate immune responses, and improve precision in cancer therapy

Beyond classical endocytic receptors, recent advances have explored novel or non-classical lysosome-trafficking receptors (LTRs) to further expand the scope and applicability of LYTAC technology. These strategies address the scarcity of suitable endogenous shuttles and offer new mechanisms for spatial and temporal control over protein degradation. A key example is the repurposing of the glucose transporter glucose transporter 1 (Glut1) as an LTR. Glut1-binding glycooligomers conjugated to antibodies create Glut1-mediated lysosomal degraders (GFLDs) that exploit Glut1’s endocytic trafficking to drive selective internalization and degradation of targets like PD-L1 on triple-negative breast cancer cells. This mechanistically distinct route demonstrates that nutrient transporters can be co-opted for targeted protein clearance [73]. Similarly, integrin α3β1, a tumor-associated adhesion receptor, has been exploited as an alternative lysosomal routing pathway. Bispecific aptamer chimeras (ITGBACs) pair an ITGA3β1-binding aptamer with a second aptamer recognizing a membrane protein of interest. These nanostructures form ternary complexes that undergo integrin-mediated endocytosis and efficient lysosomal trafficking, enabling potent degradation of pathological proteins like CD71 and PTK7, which in turn triggers cell-cycle arrest, apoptosis, and tumor suppression in vivo [74]. Notably, spatiotemporal control over LYTAC activity has been achieved using engineered biological systems. For instance, an engineered bacterium was programmed with a photothermal-inducible switch to express a transferrin receptor (TfR)-directed LYTAC only upon localized light activation. A cellphone-operated photothermal system confines LYTAC induction to illuminated tumor regions, preventing systemic exposure and off-target degradation. This approach enables precise, on-demand lysosomal routing of extracellular targets, yielding potent suppression of melanoma and colon tumors without detectable toxicity, and paving the way for telemedicine-compatible, at-home cancer treatment [75]. With PD-L1 being a prime therapeutic target. An innovative approach utilizes molecular imprinting technology to create synthetic degraders. A molecularly imprinted lysosomal nanodegrader (MILND) is engineered to specifically recognize the N-terminal epitope of PD-L1. Upon binding, the MILND drives the rapid internalization and lysosomal destruction of the immune checkpoint protein. This process restores cytotoxic T-cell activity within the tumor with high specificity, minimizing off-target immune effects. In vivo, these nanomaterials achieve durable tumor growth suppression without detectable toxicity. This strategy highlights a unique “release” mechanism where the payload is not a drug but a degradative fate for the target protein itself, offering a highly precise alternative to conventional checkpoint inhibitor antibodies [76]. Collectively, non-classical LTRs—including nutrient transporters, adhesion receptors, and engineered biological systems—substantially broaden the lysosome-targeting repertoire of LYTACs. These strategies introduce new opportunities for spatial and temporal control over protein degradation, thereby improving the precision and therapeutic potential of LYTAC-based interventions (Fig. 4).

### Structure- and regulation-guided LYTAC strategies

Beyond receptor selection, the structural and regulatory design of LYTACs critically shapes their degradation behavior and therapeutic potential by influencing stability, intracellular trafficking, and target engagement. In this context, advances in covalent engineering, stimulus-responsive design, multivalency, and nanomaterial scaffolding have provided additional layers of control over lysosome-directed protein degradation, enabling finer modulation of degradation kinetics, spatial localization, and functional outcomes.

#### Covalently modified LYTAC

Covalent modification strategies enhance LYTAC stability and efficacy by enabling irreversible target engagement. This approach mitigates dissociation kinetics and prolongs degradation activity. For instance, aptamer-based LYTACs have been covalently engineered with diazirine and arylsulfonyl fluoride warheads, allowing proximity-enabled cross-linking to membrane proteins like PTK7 or c-Met. This irreversible binding markedly improves biostability and drives sustained lysosomal clearance, enhancing natural killer cell cytotoxicity beyond non-covalent platforms [77]. Additionally, a distinct strategy employs covalent membrane protein aggregate-targeting chimeras (CMPATACs). These molecules form irreversible bonds with target proteins, inducing their aggregation on the cell surface. These large aggregates are subsequently internalized and routed to lysosomes via bulk endocytic pathways, bypassing the need for specific lysosome-trafficking receptors. This mechanism enables potent degradation of diverse targets, including traditionally “undruggable” proteins like alkaline phosphatase [78]. Similarly, peptide-based covalent platforms like Pep-TAC utilize flexible aryl-sulfonyl fluoride warheads to cross-link targets and recruit the TfR for lysosomal delivery. Notably, Pep-TACs demonstrate enhanced activity in acidic TME and can cross the blood-brain barrier, enabling effective degradation of PD-L1 in intracranial tumors [79]. In summary, covalent LYTACs–spanning aptamer, small-molecule, and peptide scaffolds–offer improved pharmacokinetic properties and sustained activity, representing a promising platform for further preclinical optimization and potential future clinical exploration.

#### Photocontrolled/Programmable LYTAC

Spatiotemporal control over LYTAC activity is achieved through photocontrolled systems, which confine protein degradation to specific sites and times, thereby minimizing off-target effects. A prominent example involves bispecific DNA aptamer chimeras embedded with near-infrared (NIR)-responsive chromophores. These phototriggered LYTACs bind both a target (e.g. PTK7) and a lysosome-trafficking receptor. Upon NIR irradiation at the tumor site, the chromophore generates localized oxidative stress, which further modulates autophagy and amplifies the lysosomal degradation cascade. This combined effect results in potent, localized tumor suppression with minimal systemic toxicity [80]. In addition, inorganic nanomaterials like Pd₆L₈-based hexagonal nanoplates loaded with indocyanine green (ICG) exhibit preferential lysosomal localization and generate a burst of singlet oxygen upon NIR exposure, causing precise lysosomal photodamage and tumor cell death [81]. It should be noted that the applicability of photo-controlled LYTACs is inherently limited by the restricted tissue penetration of light, which confines their use primarily to superficial tumors or those accessible through endoscopic or intraoperative illumination. In addition to penetration depth, achieving uniform and controllable light exposure at the tumor site remains challenging, particularly in heterogeneous tissues, which may result in incomplete or spatially uneven target degradation. While emerging approaches such as upconversion nanoparticles, X-ray-activated systems, and NIR-II-responsive platforms may help extend activation to deeper tissues, these strategies remain under active development and add further complexity to system design. Collectively, these photocontrolled platforms exemplify how external stimuli can be integrated into LYTAC design to achieve on-demand, spatially precise protein degradation, enhancing both safety and therapeutic precision.

#### Multivalent/Logic-responsive LYTAC

The engineering of multivalent and logic-responsive architectures introduces sophisticated control over LYTAC avidity, selectivity, and conditional activation, enabling precise degradation within complex biological environments. For instance, multivalent aptamer assemblies are designed to co-engage extracellular TGFβ and lysosomal trafficking receptors, markedly enhancing binding avidity to drive efficient cytokine clearance. This enforced depletion disrupts TGFβ-driven stromal activation and reprograms the immunosuppressive TME, thereby sensitizing tumors to chemotherapy and immunotherapy [82]. Parallel strategies focus on directly harnessing endogenous cellular machinery to ensure universal lysosomal routing. One approach involves grafting intrinsic lysosomal sorting signals—like the YXXØ motif from LAMP-2a—onto DNA aptamers, creating “signal aptamers” that degrade targets such as PTK7 independent of classical lysosome-trafficking receptors [83]. Similarly, a more versatile protein-based platform, SignalTACs, fuses the cation-independent mannose-6-phosphate receptor (CI-M6PR) endolysosomal sorting signal to antibodies or nanobodies, driving potent degradation of diverse targets including HER2 and PD-L1 by co-opting the cell’s intrinsic vesicular sorting apparatus [84].

Furthermore, computational design has yielded advanced modules like EndoTags, which are programmable peptide motifs that activate receptor-specific endocytosis. When fused to binders, EndoTags enable tissue-selective degradation and allow for complex functionalities such as genetic encodability and logic-gated secretion from engineered cells [85]. Lysosome-targeted logic-gated systems offer a strategy for more selective therapeutic intervention with reduced off-target effects. A representative example is Logic-TAC, a duplex DNA structure that regulates LYTAC activation through cell membrane-guided DNA logic computation. This system remains inactive in circulation, as the POI/MUC1 and LTR/IGF2R recognition regions are blocked by LE1/LE2, and becomes activated in response to dual inputs (EpCAM and MUC1) at the tumor site, thereby exposing functional domains and enabling selective lysosomal degradation of MUC1 in MCF-7 cells. In vivo, intravenous administration of Logic-TAC (1 µM) in combination with gemcitabine resulted in significant tumor growth inhibition in MCF-7 models, with a tumor-specific Cy5 signal of 85.7%, a blood half-life of 11.6 min, and no observable systemic toxicity [86]. Extending this concept, a split-deliver-click PROTAC platform employs an “AND” logic gate activated by dual lysosomal proteases (legumain and cathepsin B). In this system, PROTAC precursors (JQ1-DBCO and POMA-N₃), encapsulated within enzyme-responsive nanoparticles, are released in tumor lysosomes and subsequently reconstitute active PROTACs via SPAAC click chemistry. In vivo, intravenous administration of Leg@J-DBCO (10 mg/kg) and CTB@P-N₃ (10 mg/kg) every 2 days for 8 days achieved 78.7% tumor inhibition in HeLa models, accompanied by selective BRD4 degradation and favorable biosafety profiles [87]. In addition, the Gi-DR DNA nanorobot incorporates a YES-AND circuit responsive to extracellular K⁺ in the TME and intra-lysosomal H⁺ (pH ~5.0). K⁺ triggers G-quadruplex-mediated transmembrane delivery, while H⁺ induces i-motif-driven self-assembly into lysosome-retained fibrous aggregates, leading to lysosomal disruption and increased ROS generation. In vivo, Gi-DR loaded with GSH-responsive LDCs (camptothecin) achieved approximately 90% tumor inhibition in HeLa models, with tumor-selective accumulation (peak at 4 h) and no detectable off-target toxicity [88]. Collectively, these systems, based on DNA strand displacement, dual-enzyme activation, or ion/pH-responsive cascades, illustrate the feasibility of logic-inspired, condition-dependent lysosomal targeting in vivo. While further validation is required to fully establish the extent of logic-gated control under physiological conditions, these studies provide supportive evidence for the potential of this approach.

In summary, the integration of multivalency, Boolean logic, and engineered endolysosomal recruitment represents a significant leap forward, granting LYTACs enhanced potency, exceptional cellular precision, and a broadly applicable mechanism to overcome variable receptor expression.

#### Nanostructure-enabled LYTAC platforms

Engineered nanomaterial scaffolds can serve as versatile and multifunctional frameworks for LYTAC assembly, providing enhanced target organization, programmable responsiveness, and opportunities for integrating synergistic therapeutic functions. In this context, programmable nucleic acid nanostructures have emerged as a particularly powerful platform. For example, pH-responsive DNA-origami devices, termed intelligent modular LYTACs (IMTACs), remain inert in circulation but selectively activate within the acidic TME. Upon activation, they engage tumor-enriched receptors to drive the cooperative degradation of target pairs like EGFR and PD-L1, eliciting profound tumor suppression [89]. Similarly, circular DNA scaffolds serve as templates for printing multivalent nanobody-targeting chimeras (mNbTACs). These assemblies achieve high-avidity target binding and recruit scavenger receptors for efficient lysosomal routing, while their modular design also allows co-delivery of chemotherapeutic agents such as doxorubicin to induce immunogenic cell death [90]. In parallel, inorganic nanoparticles offer distinct physicochemical advantages. Gold nanoparticles functionalized with hypervalent bispecific aptamer chimeras (AuNP-APTACs) potently degrade the drug-efflux transporter ABCG2 on resistant cancer cells, restoring intracellular chemotherapeutic accumulation and reversing multidrug resistance [91]. Polymeric nanomaterials further expand this multifunctionality by integrating complementary therapeutic actions into a single platform. For example, a bifunctional nanostructured LYTAC (NLTC) combines lysosomal delivery of PD-L1 with co-delivery of the antioxidant enzyme catalase. This dual action simultaneously disrupts immune checkpoint signaling and scavenges immunosuppressive ROS in the TME, thereby enhancing T-cell activation and promoting antitumor immunity [92]. It is important to note that co-delivery of chemotherapeutic agents via LYTAC platforms that direct cargo to the lysosomal pathway introduces a key design consideration. The therapeutic payload must either be active within the lysosomal compartment or incorporate endosomal escape functionality to reach cytosolic or nuclear targets. Without such design, agents requiring cytosolic or nuclear localization may become sequestered within the endolysosomal system and undergo degradation, thereby reducing their efficacy. This trade-off highlights the importance of rational payload selection. For example, lysosome-activatable prodrugs may be more suitable in this context, or LYTAC-mediated degradation can be combined with endosomal escape strategies to enable complementary rather than counterproductive effects.

#### Small-molecule-based LYTAC

Complementing large biologic and nanomaterial platforms, the development of fully small-molecule LYTACs represents a pivotal direction toward oral bioavailability and enhanced tissue penetration. These compact, bifunctional molecules are designed to hijack the autophagy-lysosome pathway by conjugating a target-binding warhead to a lysosomotropic trigger. The lead compound B3 exemplifies this strategy, linking a PD-L1-inhibitory moiety to a functional scaffold that induces autophagic engulfment. This design enables robust, small-molecule-driven clearance of transmembrane PD-L1, leading to potent reactivation of antitumor immunity and effective suppression of tumor growth in vivo [93]. Thus, small-molecule LYTACs validate a critical pharmacologic approach, demonstrating that synthetic, drug-like molecules can successfully redirect membrane proteins to lysosomes, thereby broadening the degrader arsenal with compounds amenable to traditional drug development pathways.

**Fig. 5:**
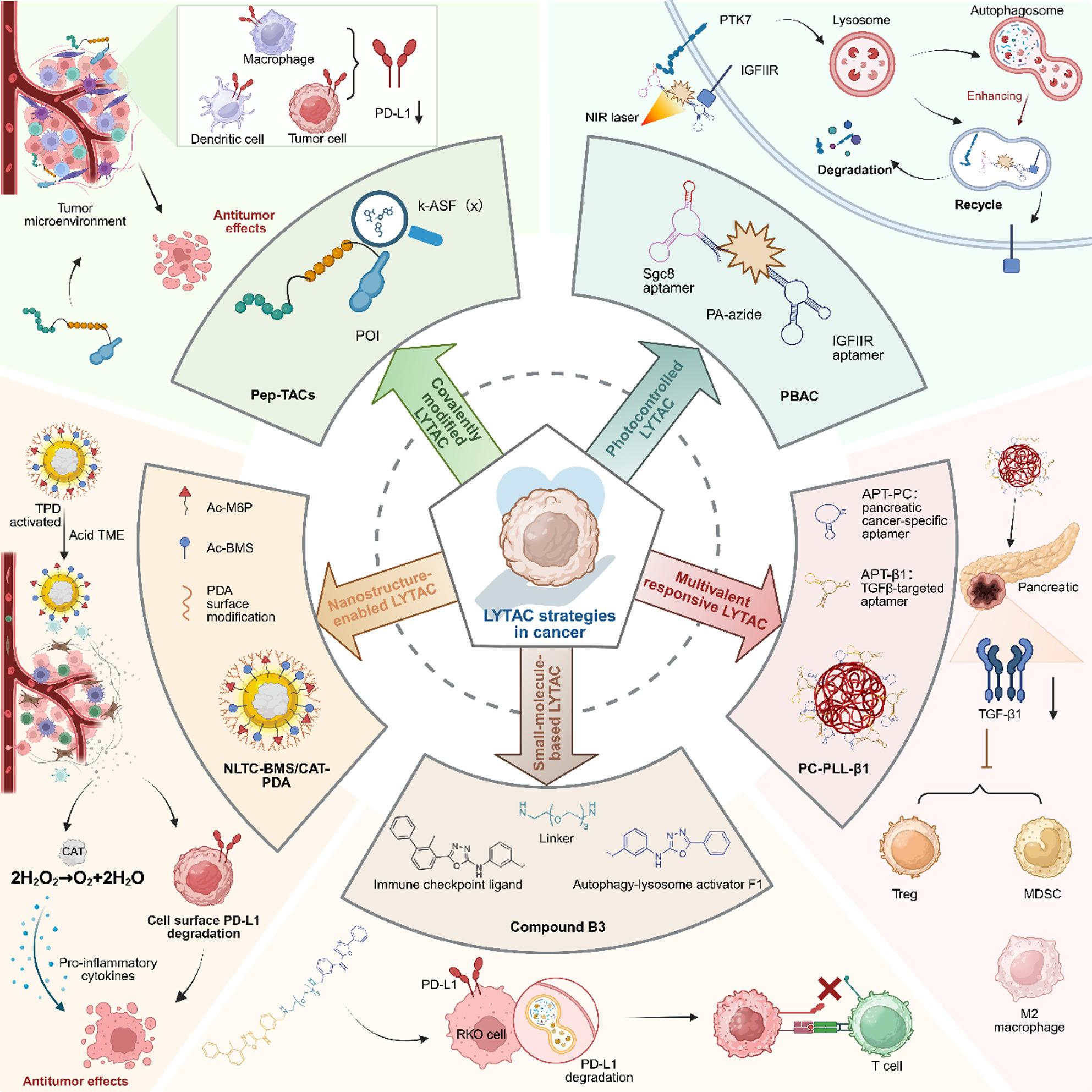
Diverse structure- and regulation-guided LYTAC platforms for controlled protein degradation. The figure illustrates five advanced LYTAC strategies beyond classical receptor engagement: (i) Covalent LYTACs (e.g., Pep-TAC), which utilize warheads like aryl-sulfonyl fluoride to form irreversible crosslinks with target proteins (e.g., PD-L1), enhancing stability and enabling transferrin receptor-mediated lysosomal delivery even across the blood-brain barrier; (ii) Photocontrolled LYTACs, where near-infrared (NIR)-responsive chromophores are integrated into bispecific aptamers, allowing spatiotemporally precise activation of degradation and autophagy modulation at tumor sites; (iii) Multivalent/Logic-responsive LYTACs, including multivalent aptamer assemblies for high-avidity cytokine clearance (e.g., TGFβ) and Boolean AND-gate DNA platforms (Logic-TAC) that degrade targets only upon sensing dual tumor-specific surface inputs, achieving exceptional cellular specificity; (iv) Small-molecule LYTACs (e.g., compound B3), which conjugate target-binding moieties to lysosomotropic triggers, enabling oral bioavailability and autophagic-lysosomal clearance of transmembrane proteins like PD-L1; (v) Nanostructure-enabled LYTACs, such as polymer-based nanoplatforms (NLTC) that co-deliver checkpoint degraders and auxiliary agents (e.g., catalase) for synergistic TME remodeling and immunotherapy. Collectively, these structural and regulatory innovations provide precise control over degradation kinetics, spatial localization, and activation logic, expanding LYTAC applicability to heterogeneous tumors and hard-to-treat microenvironments

Collectively, the expanding repertoire of LYTAC strategies underscores the modular and highly adaptable nature of lysosome-directed protein degradation in cancer. Receptor-guided approaches establish cell- and tissue-selective entry routes, while structure- and regulation-oriented designs—including covalent engagement, stimulus responsiveness, multivalency, nanostructure-enabled assembly, and small-molecule modalities—provide additional layers of control over degradation kinetics, spatial localization, and functional outcomes. Together, these advances transform LYTACs from a receptor-dependent concept into a versatile degradation platform capable of overcoming heterogeneity in receptor expression, minimizing off-target effects, and addressing multiple resistance mechanisms. As such, LYTAC-based technologies represent a promising and increasingly sophisticated therapeutic paradigm for targeting otherwise intractable membrane proteins in cancer (Fig. 5).

### Biological constraints and nanoplatform design solutions

Despite the remarkable progress in receptor-guided lysosomal degradation strategies, several inherent biological constraints still limit their clinical translation. A clearer understanding of these barriers, together with the corresponding nanoplatform design solutions, is essential for the development of robust and selective therapies. One major challenge is receptor heterogeneity and variable expression. Lysosome-trafficking receptors (LTRs), including ASGPR, IGF2R, FRα, GPC3, and HER2, often show substantial interpatient and intratumoral heterogeneity, which can lead to inconsistent degradation efficiency and the emergence of resistant cell populations with low receptor density. To mitigate this issue, nanoplatforms can be engineered to present multivalent ligands on scaffolds such as DNA origami or polymeric nanoparticles, thereby enhancing binding avidity [64]. Cooperative targeting strategies that simultaneously engage two distinct LTRs may further reduce the receptor-density threshold required for efficient internalization [94, 95]. In addition, receptor-independent degradation mechanisms, such as covalent membrane protein aggregation or the use of intrinsic lysosomal sorting signals, provide an alternative means of bypassing LTR variability altogether [84].

A second key constraint involves internalization kinetics and trafficking dynamics, which critically determine the efficiency of lysosomal delivery. Slow internalization can prolong target exposure at the cell surface, while different LTRs follow distinct intracellular itineraries; for example, ASGPR undergoes rapid recycling, whereas sortilin traffics more slowly, resulting in differences in lysosomal delivery efficiency. Several nanoplatform design strategies have been proposed to address these limitations. Tuning nanoparticle geometry and ligand density can accelerate uptake, and high-aspect-ratio nanostructures often display faster internalization [96]. Multivalent engagement that crosslinks multiple target proteins can also strengthen endocytic signaling and thereby promote more efficient uptake [97]. Moreover, photocontrolled or stimuli-responsive systems can provide spatiotemporal control over internalization, enabling better synchronization of uptake and trafficking processes [98, 99].

Another important limitation is receptor recycling and target re-emergence. If the LYTAC–target complex does not dissociate efficiently from the receptor, co-recycling may occur, thereby reducing overall degradation efficiency. At the same time, rapid receptor recycling can also alter the availability of surface LTRs. To overcome these problems, covalent LYTAC designs have been developed to irreversibly link the targeting moiety to the target protein, thereby preventing complex dissociation and promoting sustained lysosomal delivery [95, 100]. In parallel, certain nanomaterials can interfere with receptor recycling pathways and prolong LTR surface availability [96, 101]. Controlled-release nanocarriers may also support sequential or pulsatile dosing regimens, which could help maintain degradation efficiency over time.

A further challenge is off-tumor uptake and systemic toxicity. Many LTRs, such as ASGPR and IGF2R, are also expressed in normal tissues, and even tumor-associated receptors such as HER2 may be present at low levels in healthy epithelia. This raises the risk of unintended uptake outside tumors. To improve selectivity, nanoplatforms can incorporate conditional activation mechanisms. For example, logic-gated LYTACs can be designed to require dual tumor-specific inputs, such as acidic pH and a surface marker, before degradation activity is triggered [92, 94]. Tumor-selective receptor engagement provides another solution, as illustrated by CAIX-targeted Supra-LYTACs that preferentially function in hypoxic tumor regions [72]. In addition, biomimetic coatings, including cancer cell membranes, can reduce nonspecific uptake [102, 103], while prodrug-like LYTAC designs that remain inactive in circulation and become activated only within the TME may further limit systemic toxicity [92, 104].

Finally, competition with endogenous ligands may compromise therapeutic efficacy. Many LTRs naturally bind endogenous molecules, such as IGF2 for IGF2R and asialoglycoproteins for ASGPR, which can compete with synthetic LYTACs for receptor occupancy. One strategy to overcome this issue is to engineer LYTACs with ultra-high-affinity receptor-binding domains. For instance, engineered IGF2 mutants with enhanced receptor affinity but abolished mitogenic signaling have been developed to outcompete endogenous ligands [105]. Alternatively, selecting receptors with low endogenous ligand occupancy or adopting receptor-independent degradation routes may help circumvent this problem entirely [62].

In summary, receptor-guided lysosomal degradation is constrained by receptor heterogeneity, variable internalization and trafficking behavior, receptor recycling, off-tumor uptake, and endogenous ligand competition. Nonetheless, rational nanoplatform design—including multivalency, avidity tuning, conditional activation, covalent engagement, and trafficking modulation—offers multiple strategies to mitigate these limitations and improve both selectivity and therapeutic robustness.

## Comparative analysis of lysosome-centered strategies: defining use-case boundaries and major limitations

Although these lysosome-centered strategies all involve the lysosome, they do so for different therapeutic purposes. In some cases, the lysosome serves as the site of drug activation or release; in others, it acts as a barrier to cytosolic or nuclear delivery, or becomes the direct target of therapeutic intervention. LYTACs and related nanodegrader systems further use lysosomal trafficking as the endpoint for selective degradation of extracellular or membrane-associated targets [106]. These differences define distinct use-case boundaries and major limitations, indicating that these approaches should be considered as mechanistically differentiated strategies rather than parallel catalogs.

### Lysosome-triggered payload activation and release

Lysosome-triggered release is most suitable when therapeutic activity depends on drug release or activation within the acidic and enzyme-rich lysosomal environment. This strategy is particularly relevant to small molecules, prodrugs, and stimulus-responsive nanocarriers designed to improve intracellular selectivity and reduce premature systemic exposure [107]. A major advantage of this approach is that lysosomal processing can confine drug release to intracellular compartments reached after endocytic uptake, thereby improving spatial control over treatment. However, its effectiveness depends on whether lysosomal release is sufficient for action at the final site of interest. If the released drug can function within the lysosome or readily reach its intracellular target after release, this approach may be effective. By contrast, if therapeutic activity requires intact delivery of biomacromolecules to the cytosol or nucleus, lysosomal release alone is usually insufficient. In such cases, incomplete release, lysosomal degradation, or prolonged endolysosomal retention may reduce efficacy. Premature release before lysosomal trafficking may also weaken selectivity [108]. Therefore, this strategy is most appropriate for payloads whose activity is compatible with lysosomal processing or with redistribution after release.

### Endolysosomal escape

Endolysosomal escape is required when the therapeutic cargo must act outside the lysosome, especially in the cytosol or nucleus. This applies to siRNA, mRNA, gene-editing systems, proteins, and other membrane-impermeable biomacromolecules [109]. Under these conditions, the lysosome is not the site of therapeutic action, but a degradative barrier that must be bypassed before the payload loses activity. This is the key distinction from lysosome-triggered release: for biomacromolecular cargos that cannot cross membranes on their own, release within lysosomes does not ensure delivery to the relevant intracellular target. The main limitation of this strategy lies in the need to balance escape efficiency with cellular safety. Insufficient escape results in lysosomal degradation and poor functional delivery, whereas excessive membrane disruption may cause nonspecific toxicity, inflammatory responses, or unintended cell death. In addition, escape efficiency must be matched with payload stability and release kinetics, particularly for biologics that are structurally fragile and highly dose-dependent [110]. Endolysosomal escape is therefore essential when extra-lysosomal delivery is required, but it is not necessarily desirable in systems where lysosomal retention or lysosomal activity is itself therapeutically useful.

### Lysosomal function modulation

Direct modulation of lysosomal function differs from the two strategies above because the lysosome itself becomes the therapeutic target. This approach is most relevant when tumor cells depend on lysosome-related processes, such as pH homeostasis, autophagic flux, lysosomal membrane integrity, or lysosome-associated signaling pathways. In these settings, disruption of lysosomal function may directly induce tumor cell death, interfere with stress adaptation, or increase sensitivity to other treatments [111]. Compared with release- or escape-based approaches, lysosomal function modulation often produces broader biological effects. This may be advantageous when several survival pathways need to be affected at the same time, but it also increases mechanistic complexity. Because lysosomal activity is closely linked to metabolism, proteostasis, and immune regulation, its disruption may trigger compensatory responses or produce context-dependent effects in different tumor types [112]. In addition, prolonged or systemic interference with lysosomal pathways may affect normal tissues in which lysosomal turnover and autophagy are physiologically important. This strategy is therefore most suitable when lysosomal dependence itself represents a therapeutic vulnerability, but its selectivity and downstream effects require careful evaluation.

### LYTACs and related nanodegrader strategies

LYTACs and related nanodegrader platforms are most suitable when the therapeutic goal is the selective depletion of extracellular or membrane-associated pathogenic proteins. Their main advantage is that they direct the target to lysosomal degradation rather than simply blocking its activity, which may provide a more sustained effect in settings driven by persistent receptor signaling, stromal mediators, or immune checkpoint expression [79]. In this context, the lysosome serves as the final site of target elimination rather than as a release compartment or a delivery barrier. This also distinguishes LYTAC-type systems from intracellular delivery strategies, which are designed to transport therapeutic cargo to a site of action within the cell [57]. The main limitations of this strategy arise from its dependence on target accessibility and receptor-mediated trafficking. Effective degradation requires not only target binding, but also recruitment of a trafficking receptor capable of directing the complex into lysosomes. Reduced receptor expression, heterogeneous receptor distribution, inefficient internalization, competition from endogenous ligands, or diversion into recycling pathways may all decrease degradation efficiency. In addition, this strategy is not readily suited to cytosolic or nuclear targets. LYTAC-type systems are therefore particularly useful for accessible extracellular or membrane proteins, but they do not replace intracellular delivery approaches.

## Lysosome-targeted therapeutic modalities for cancer treatment

Beyond serving as a trafficking hub or a site for cargo processing, the lysosome has emerged as an actionable therapeutic locus for direct cancer treatment [56]. A growing class of lysosome-targeted therapeutic modalities intentionally concentrates externally activated or catalytically active agents within this organelle to induce localized cytotoxicity. By exploiting the lysosome’s acidic milieu, enzyme-rich lumen, and membrane vulnerability, these strategies convert physical stimuli or chemical reactions into potent intracellular killing mechanisms. In this context, lysosomal targeting is not merely a delivery consideration but a central determinant of therapeutic efficacy, enabling spatially confined damage that amplifies tumor cell death while limiting systemic toxicity.

### Lysosome-targeted sonodynamic therapy

Sonodynamic therapy (SDT) employs ultrasound to activate tumor-localized sonosensitizers, thereby generating cytotoxic ROS with deep tissue penetration and high spatial precision [113]. Although the underlying mechanisms of SDT are well established, recent studies have increasingly focused on improving therapeutic performance through advanced delivery systems that exploit subcellular targeting, particularly lysosomal accumulation and acidic microenvironment responsiveness. These strategies have provided additional opportunities to enhance antitumor efficacy in vivo. For example, a polythiophene derivative (PT2) encapsulated with folic acid-functionalized polyethylene glycol (DSPE-PEG-FA) was formulated into PDPF nanoparticles as a sonosensitizer with high ultrasound stability. This nanoplatform was designed to preferentially accumulate in both lysosomes and the plasma membrane of cancer cells, thereby enabling in situ ROS-mediated organelle damage upon ultrasound activation. In a 4T1 murine breast cancer model, intravenous administration of PDPF nanoparticles (43 µM, 200 µL), followed by ultrasound irradiation (1.75 W/cm², 10 min), significantly inhibited tumor growth. After 14 days, tumor volumes in the treatment group were markedly reduced compared with those in the control groups, while no obvious changes in body weight were observed. Histological analyses, including H&E, Ki67, and TUNEL staining, further confirmed extensive apoptosis and reduced proliferative activity in tumor tissues, supporting the antitumor efficacy of this lysosome-accumulative sonosensitizer [114]. Another strategy utilized tumor cell-derived exosomes as homotypic delivery vehicles for the porphyrin sonosensitizer sinoporphyrin sodium (DVDMS), resulting in the exosomal formulation EXO-DVDMS. After endocytic uptake by tumor cells, EXO-DVDMS was trafficked to lysosomes, where the acidic microenvironment, together with ultrasound exposure, promoted controlled DVDMS release and redistribution. This process enhanced ROS generation and activated multiple cell death pathways. In a 4T1 murine breast cancer model, intravenous administration of EXO-DVDMS (2 mg/kg), followed by sequential ultrasound irradiation at 24 h post-injection (guiding US1: 2 W, 3 min; therapeutic US2: 3 W, 3 min), achieved a tumor growth inhibition rate of 74.88%. This treatment also produced a pronounced anti-metastatic effect, reducing the average number of pulmonary metastatic nodules to 1.5 ± 0.71, compared with 130.5 in the control group. Safety evaluations, including body weight monitoring, histological examination of major organs, and serum biochemical analyses of ALT, AST, and BUN, indicated good biocompatibility of this exosome-based sonosensitizing platform [115]. In addition to single-modality SDT, an ultrasound-actuated ion homeostasis perturbation strategy was developed using PCCa, a nanoplatform composed of a porphyrinic metal-organic framework (PCN) core coated with a CaCO₃ shell. Following endocytosis, PCCa was trafficked to lysosomes, where the acidic environment triggered CaCO₃ decomposition and rapid Ca²⁺ release. At the same time, ultrasound-activated PCN generated ROS, which impaired cellular Ca²⁺ buffering capacity and thereby intensified oxidative stress and calcium dysregulation. In a 4T1 murine breast cancer model, intravenous administration of PCCa (10 mg/kg), followed by low-intensity focused ultrasound irradiation (1.5 W/cm², 10 min), significantly suppressed primary tumor growth and reduced lung metastasis. Notably, this effect was further enhanced when PCCa-mediated SDT was combined with anti-PD-1 immunotherapy, resulting in superior tumor control. Biosafety assessments, including body weight monitoring, histological analysis of major organs, and serum biochemical measurements of ALT, AST, and BUN, revealed no overt toxicity [116]. Collectively, these studies illustrate how lysosomal targeting, exosome-mediated delivery, and lysosome-responsive ion interference can improve the performance of SDT nanoplatforms. By integrating subcellular targeting with tumor-responsive design, these approaches broaden the therapeutic potential of SDT for cancer treatment.

### Lysosome-targeted photodynamic therapy

PDT uses light-activated photosensitizers to generate lethal ROS within specific subcellular compartments, and lysosomal targeting has increasingly been explored as a means to enhance therapeutic efficacy and modulate immune responses [117]. Addressing the critical limitation of tumor hypoxia in PDT, a biomimetic protein self-assembly strategy has been developed using NIR Ag₂S quantum dots as structural and imaging guides. Through pH-dependent electrostatic interactions, catalase modified with the photosensitizer chlorin e6 (Ce6) was assembled with quantum dots and loaded with oxaliplatin to form a multifunctional nanoplatform. This system enabled TME-responsive disassembly for sustained drug release, while catalase-mediated oxygen generation significantly alleviated hypoxia to enhance PDT efficacy. In colorectal tumor models, the platform achieved remarkable synergistic antitumor effects under imaging guidance, demonstrating how enzyme-assisted oxygen self-supply can potentiate lysosome-localized PDT [118]. Another representative strategy involves semiconducting polymer nanoparticles (SPSS NPs) engineered with surface self-assembled peptide antagonists that form β-sheet-like multivalent structures. In addition to serving as efficient PDT agents that induce immunogenic cell death (ICD) through robust ROS generation, these nanoparticles also promote the internalization and lysosomal degradation of PD-L1. In a CT26 tumor-bearing mouse model, intravenous administration of SPSS NPs (200 µL, 1 mg/mL), followed by light irradiation (0.3 W/cm², 10 min) at 12 h post-injection, reduced the mean tumor volume to approximately 17 mm³ by day 19. Moreover, 60% of treated mice achieved complete tumor eradication and survived for more than 55 days, while body weight remained stable throughout treatment, indicating good tolerability [119]. To further regulate the immunological consequences of PDT-induced cell death, positively charged carbon dots (PCDs) were developed with time-dependent subcellular redistribution properties. Early localization at the plasma membrane (0.5 h post-injection) favored GSDME-mediated pyroptosis upon light activation, whereas later accumulation in lysosomes (6 h post-injection) shifted the mode of cell death toward apoptosis. Notably, membrane-targeted activation induced stronger ICD than lysosome-targeted activation. In a 4T1 tumor-bearing mouse model, intratumoral injection of PCDs (2 mg/kg), followed by irradiation (577 nm, 100 mW/cm², 8 min) at 0.5 h post-injection, achieved nearly complete suppression of primary tumor growth and effectively inhibited contralateral tumor growth in a rechallenge model. This effect was markedly superior to that observed with irradiation at 6 h post-injection or in the control groups. Body weight remained stable in all groups, and histological examination of major organs further supported the biosafety of this treatment [120]. Further improving the precision of PDT, intelligent DNA nanospheres (NS) have been designed with i-motif structures capable of decoding subtle pH differences along the endocytic pathway. This design enables selective activation in early endosomes (NSₑₑ) to induce GSDME-mediated pyroptosis or in lysosomes (NSₗ_γ_) to trigger cathepsin B-dependent apoptosis. In an MCF-7 tumor-bearing mouse model, intravenous administration of aptamer (AS1411)-targeted NSₑₑ, followed by irradiation (100 mW/cm², 5 min) at 0.5 h post-injection, achieved a tumor eradication rate of 82.7%, which was significantly higher than that of lysosome-activated NSₗ_γ_ (49.2%) or non-targeted NS (18.4%). Histological analysis of tumor tissues confirmed pyroptosis in the NSₑₑ group, whereas hemolysis assays (< 5% hemolysis) and H&E staining of major organs, including the heart, liver, spleen, lung, and kidney, showed no overt toxicity [121]. In addition to regulating cell death pathways, lysosome-targeted PDT has also been used to remodel the tumor immune microenvironment. For example, a type I/II photosensitizer with aggregation-induced emission (AIE) properties, CPBPDPN-TPA, was shown to selectively accumulate in lysosomes. Upon white light irradiation, it generated substantial levels of ROS, including •OH and ¹O₂, thereby inducing apoptosis and ICD through disruption of the autophagy–lysosome pathway. This process further promoted the release of damage-associated molecular patterns (DAMPs) and activation of the STING pathway, contributing to reprogramming of the immunosuppressive TME. In a CT2A subcutaneous tumor model, intratumoral injection of CPBPDPN-TPA (50 µL, 1 mM), followed by irradiation (150 mW/cm², 15 min) at 8 h post-injection, significantly inhibited tumor growth, with the strongest therapeutic effect observed when combined with anti-PD-1 treatment. H&E and TUNEL staining revealed extensive tumor necrosis, while histological examination of major organs showed no detectable damage, supporting its favorable biosafety profile [122].

Collectively, these studies show that lysosome-targeted PDT has progressed beyond a simple cytotoxic strategy and can now be designed to regulate the mode of cell death and strengthen antitumor immune responses, thereby expanding its therapeutic potential in cancer treatment. Despite its well-established efficacy, the clinical utility of PDT is constrained by the shallow penetration depth of activating light, limiting its effectiveness to superficial or optically accessible tumors. Moreover, the dependence on oxygen for ROS generation introduces an additional layer of limitation in hypoxic tumor regions, where PDT efficacy may be compromised. To address these challenges, strategies such as two-photon excitation, upconversion nanoparticles that convert NIR light to visible emission, and X-ray-activated photosensitizers have been explored to improve tissue penetration and expand therapeutic reach, although these approaches require further validation in clinically relevant settings.

### Lysosome-focused photothermal therapy and magnetic hyperthermia

Thermal therapies targeting the lysosome exploit its vulnerability to heat and ROS to achieve selective tumor ablation. One representative approach is magnetic intra-lysosomal hyperthermia (MILH), which uses gastrin-grafted iron oxide nanoparticles to target CCK2R-overexpressing tumor cells and accumulate in lysosomes through receptor-mediated internalization. Upon exposure to an alternating magnetic field (275 kHz, 40 mT, 2 h), these nanoparticles generate localized heat within lysosomes, thereby enhancing ROS production through the Fenton reaction. This process induces lysosomal lipid peroxidation, membrane permeabilization, and release of cathepsin B, ultimately triggering caspase-1-dependent pyroptosis without activation of apoptotic caspase-3. Notably, MILH can also be combined with doxorubicin to achieve synergistic antitumor effects by engaging complementary cell death pathways, namely MILH-induced caspase-1-dependent pyroptosis and doxorubicin-mediated caspase-3-dependent apoptosis. These findings support MILH as a promising lysosome-directed magnetic hyperthermia strategy, either as a standalone treatment or as a chemosensitizing modality [123, 124].

Related advances in PTT have focused on organic agents engineered for dual targeting of tumor cells and lysosomes. For example, the amphiphilic photothermal agent M-PT-RGD self-assembles into nanoparticles (50 ± 15 nm) bearing both cRGD, a tumor-targeting ligand, and morpholine, a lysosome-targeting moiety. Upon 635 nm laser irradiation (0.5 W/cm², 5 min), this nanoplatform exhibited a photothermal conversion efficiency of 43.29% and generated localized hyperthermia sufficient to disrupt lysosomal integrity, as confirmed by acridine orange staining. In a 4T1 tumor-bearing mouse model, intravenous administration of M-PT-RGD followed by laser irradiation (0.5 W/cm², 10 min) at 12 h post-injection, corresponding to the peak of tumor accumulation, resulted in complete tumor regression within 16 days without recurrence. Biosafety evaluations, including body weight monitoring, H&E staining of major organs, and blood biochemical analysis, showed no evident systemic toxicity [125]. Another lysosome-directed photothermal strategy employed gold nanostars densely functionalized with anti-HER2 aptamers. These nanostructures undergo HER2-mediated endocytosis and are subsequently trafficked to lysosomes, where the acidic and protease-rich environment accelerates degradation of the oncogenic HER2 receptor. This process suppresses HER2-driven proliferative signaling and induces cell cycle arrest, further illustrating how lysosomal trafficking can be coupled to therapeutic intervention in tumor cells [126]. Beyond synthetic ligand-based targeting, biomimetic approaches have emerged as a powerful strategy to enhance lysosome-focused PTT, a recent study developed a tumor cell membrane-encapsulated nanodelivery system incorporating Ag₂S quantum dots as NIR-II photothermal agents, chemotherapeutic drugs, and PD-L1 inhibitors. The homologous targeting properties conferred by the cancer cell membrane enabled efficient lysosomal accumulation and tumor-specific delivery. Upon NIR-II irradiation, the platform induced pronounced photothermal effects while co-activating immunogenic cell death, which synergized with PD-L1 blockade to achieve a 56.5% immune checkpoint inhibition rate and reduce lung metastatic nodules by 51.2% in a triple-negative breast cancer model. This work illustrates how biomimetic coating can integrate lysosomal photothermal action with immune modulation to address metastatic disease [127]. Expanding this strategy further, a human serum albumin-based nanoplatform (HSA@ICG/siRNA NPs) was developed for combined PTT and HSP70 gene silencing. This system co-delivers ICG and HSP70-targeting siRNA to address the thermoresistance commonly associated with PTT. Upon 808 nm laser irradiation, the nanoparticles generate mild hyperthermia (42–46 °C) to induce direct photothermal damage, while also promoting lysosomal escape of siRNA into the cytoplasm, thereby enhancing gene silencing efficiency. In a 4T1 tumor-bearing mouse model, intravenous administration of HSA@ICG/siRNA NPs (3 mg/kg ICG, 13.3 µg/kg siRNA), followed by 808 nm laser irradiation (initially 1.91 W/cm² for 3 min and then 0.76 W/cm² for 3 min per session), achieved a tumor inhibition rate of 71.26% ± 7.92%, which was significantly greater than that of PTT alone (50.00% ± 9.16%). This combination treatment also reduced HSP70 mRNA expression to 17.2% and decreased HSP70 protein levels by 65%. No obvious systemic toxicity was observed, as indicated by stable body weight and unremarkable H&E staining of major organs [128].

Similar to PDT, conventional PTT is restricted by limited light penetration, which constrains its application to superficial or accessible tumors. In addition, achieving homogeneous thermal distribution within deeper tumor regions remains difficult, potentially leading to suboptimal ablation or damage to surrounding tissues. Efforts to improve the depth of activation include the development of NIR-II-absorbing agents and the use of fiber-optic or interstitial light delivery techniques. Notably, alternative modalities such as magnetic hyperthermia, which rely on externally applied magnetic fields rather than light, can circumvent these optical limitations and may offer complementary advantages for treating deeply located tumors.

### Lysosome-amplified chemodynamic therapy

CDT uses transition metal ions to catalyze the conversion of endogenous hydrogen peroxide into highly cytotoxic hydroxyl radicals within tumors, and lysosomal localization can markedly enhance this process by concentrating the relevant reactants. One representative example is the biodegradable MnS@HA-DOX nanocluster, which was fabricated by biomineralization using hyaluronic acid (HA) as both a CD44-targeting scaffold and a carrier for doxorubicin (DOX). After intravenous administration (DOX-equivalent dose, 5 mg/kg), CD44-mediated endocytosis and subsequent trafficking to endo/lysosomes (pH ~4.5–5.5) triggered acid-dependent disassembly of the nanocluster, leading to the release of Mn²⁺, H₂S, and DOX. In this system, Mn²⁺ catalyzed Fenton-like generation of hydroxyl radicals, H₂S increased intracellular H₂O₂ levels by suppressing catalase activity, and DOX further amplified ROS production through NADPH oxidase activation. Together, these effects induced DNA double-strand breaks, G2/M cell-cycle arrest, and apoptosis. In vitro, this nanoplatform induced 77.4% late apoptosis in MCF-7 cells. In vivo, it significantly inhibited tumor growth in an MCF-7 xenograft model over 14 days and reduced the area of lung metastatic nodules to 1.78%. Its biosafety was supported by hemolysis below 5%, stable hematological parameters, and the absence of obvious damage in major organs on H&E staining [129]. Expanding the scope of lysosome-enhanced CDT, a NIR-responsive Janus nanomotor (Z@P-F) was developed using a zinc peroxide (ZnO₂) core asymmetrically coated with polydopamine (PDA), enabling both self-thermophoretic motion and synergistic ferroptosis induction. Following endocytosis and lysosomal accumulation (pH ~5.0–6.0), the acidic environment promoted the release of Zn²⁺ and H₂O₂ from ZnO₂, together with Fe²⁺ generated through PDA chelation, thereby sustaining the Fenton reaction. Under 808 nm laser irradiation (1.5 W/cm²), the asymmetric PDA coating generated a thermal gradient that drove nanomotor motion, resulting in enhanced diffusion that was 1.87-fold greater than Brownian motion. This property improved tumor penetration, reaching a penetration efficiency of 65.04 ± 1.73% at a depth of 100 μm, and also facilitated lysosomal escape. In a 4T1 tumor-bearing mouse model, a single intravenous injection of Z@P-F (5 mg/kg ZnO₂, day 0), followed by NIR irradiation (1.5 W/cm², 3 min), reduced tumor volume by 98.92 ± 0.28%, significantly outperforming the non-motile control group. Mechanistically, the treatment induced pronounced ferroptosis, as evidenced by downregulation of GPX4 and SLC7A11 together with increased lipid peroxidation. No obvious systemic toxicity was observed, as indicated by stable body weight, normal blood biochemical parameters, and unremarkable H&E staining of major organs [130].

**Fig. 6:**
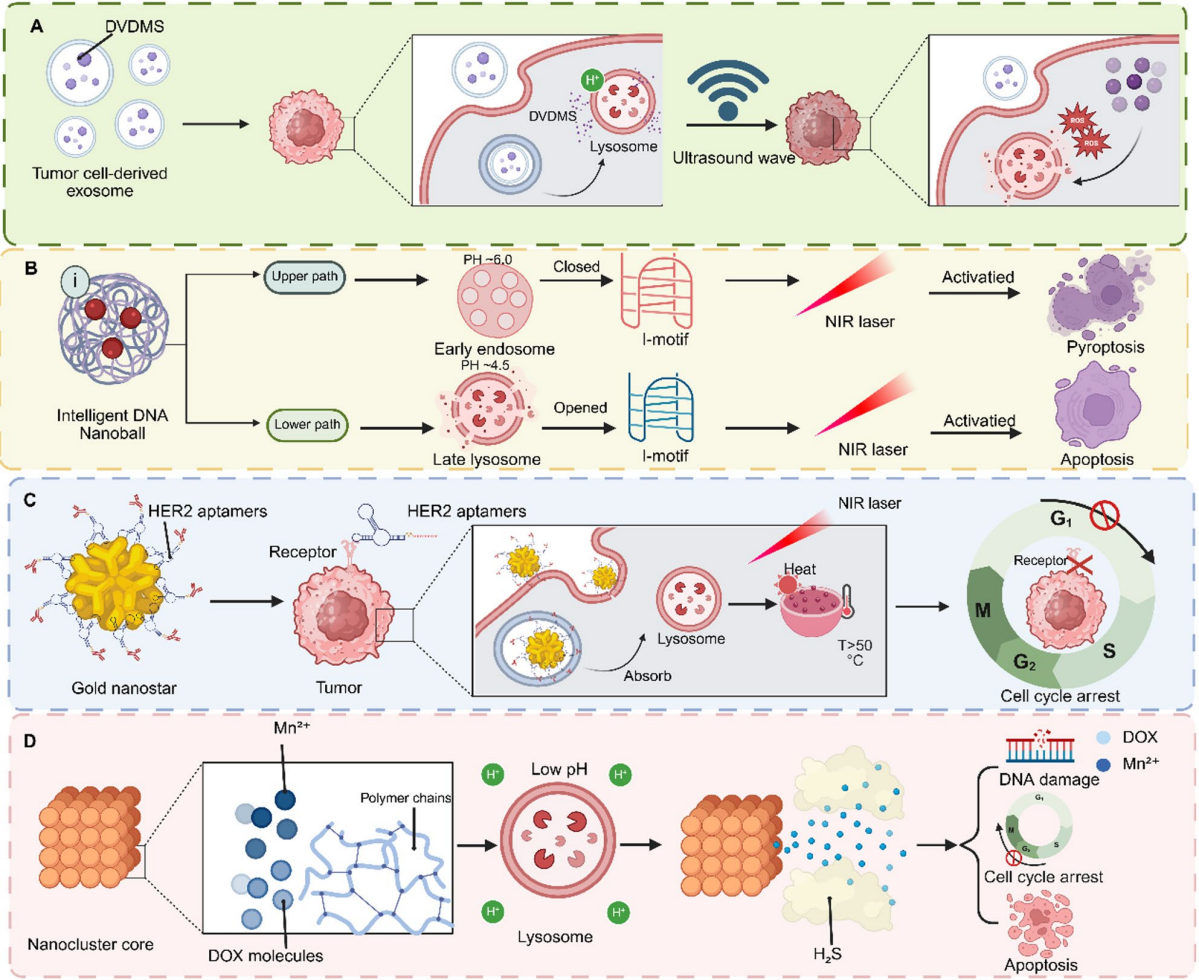
Lysosome-targeted multimodal nanotherapeutics. The lysosome serves as a strategic hub for synergistic nanotherapeutics, where spatially confined activation of diverse physical and chemical stimuli can amplify therapeutic specificity and efficacy. Four representative mechanisms are shown: (**A**) Sonodynamic therapy employs exosome-delivered sonosensitizers (DVDMS) that accumulate in lysosomes; ultrasound triggers a localized ROS burst, leading to lysosomal membrane permeabilization and apoptosis. (**B**) PDT uses pH-sensing i-motif DNA nanospheres that differentiate between endosomal compartments: activation in early endosomes (pH ~6.0) induces pyroptosis, while in lysosomes (pH ~4.5) it triggers apoptosis. (**C**) Photothermal therapy leverages HER2 aptamer-conjugated gold nanostars that traffic to lysosomes; NIR irradiation generates localized hyperthermia (> 50 °C), degrading HER2 and arresting the cell cycle. (**D**) Chemodynamic therapy relies on acid-degradable MnS@HA-DOX nanoclusters that dissociate in lysosomes, releasing Mn²⁺ for Fenton-like ROS generation, H₂S gas, and doxorubicin to synergistically induce DNA damage and apoptosis. Collectively, these lysosome-targeted strategies exemplify how nanomaterials can integrate physical activation (ultrasound, light) with biochemical reactions (ROS, enzyme degradation) within a defined organelle to achieve multimodal cancer therapy with enhanced precision and reduced off-target effects

The diverse therapeutic approaches under the paradigm of lysosome-targeted lethal delivery converge on a unifying principle: the strategic exploitation of the lysosome as a vulnerable and programmable compartment for inducing selective cancer cell death. Whether through externally applied stimuli (ultrasound, light, magnetic fields) or intrinsic catalytic reactions, these modalities are designed to concentrate cytotoxic agents—sonosensitizers, photosensitizers, thermal converters, or Fenton catalysts—within the acidic, enzyme-rich lysosomal lumen. This localization enables the potent, spatially confined generation of destructive forces, such as ROS bursts, hyperthermia, or radical cascades, which trigger organelle rupture, biomolecular damage, and the initiation of specific death pathways (apoptosis, pyroptosis, or lysosome-dependent necrosis). By confining the lethal payload to this key organelle, these strategies significantly enhance therapeutic specificity and efficacy against tumors while minimizing off-target damage to healthy tissues, thereby establishing lysosome-focused delivery as a refined and powerful framework in precision oncology (Fig. 6).

## Challenges and future perspectives

Despite the remarkable progress of nanomaterial-based lysosome-targeted strategies in cancer therapy, substantial challenges remain along the path from conceptual innovation to clinical translation. These challenges span technological feasibility, biological complexity, and manufacturing scalability, and must be addressed through coordinated advances across disciplines.

At the foundational level, our understanding of the cellular response networks triggered by lysosome-targeted interventions remains limited. Most studies focus on immediate therapeutic endpoints, such as protein degradation efficiency or cancer cell death, while compensatory signaling pathways and adaptive responses are often overlooked. For example, enforced degradation of PD-L1 may activate interferon signaling, leading to the upregulation of alternative immune checkpoint molecules [131]. Similarly, disruption of lysosomal membrane integrity may induce protective responses such as selective autophagy. These complex feedback mechanisms complicate outcome prediction and may undermine long-term efficacy. To address this gap, integrative multi-omics approaches—including transcriptomics, proteomics, and metabolomics—combined with single-cell analyses will be required to generate comprehensive molecular response maps following lysosomal intervention [132]. Advanced disease models, such as patient-derived organoids and humanized animal systems, will further improve physiological relevance [133]. In parallel, computational modeling and artificial intelligence–based data integration are expected to play an increasingly important role in identifying key regulatory nodes and predictive biomarkers, thereby guiding rational therapeutic design.

Building upon this biological complexity, another layer of intricacy arises from the adaptive responses that cancer cells mount against lysosome-targeted interventions. Prolonged disruption of lysosomal function can trigger compensatory programs, including autophagy rewiring, enhanced lysosomal biogenesis driven by transcription factor EB (TFEB) nuclear translocation, and activation of stress-response pathways such as the unfolded protein response and oxidative stress adaptation. These processes are often coordinated through integrated nutrient-sensing and stress-signaling networks, enabling tumor cells to rebalance protein and organelle turnover, maintain redox homeostasis, and preserve metabolic flexibility under lysosomal stress [134, 135]. In particular, increased lysosomal biogenesis may expand degradative capacity, while autophagy rewiring can redirect intracellular recycling toward alternative routes that support survival. Importantly, such adaptive programs can dynamically reshape cellular dependencies, allowing cancer cells to tolerate sustained lysosomal perturbation and thereby diminishing therapeutic efficacy over time [136]. These considerations highlight the need for rational combination strategies. One potential approach is to concurrently limit lysosomal biogenesis, for example through small-molecule modulators of TFEB, to restrain the expansion of the lysosomal compartment [137]. In parallel, combining lysosome-targeted nanotherapies with immune checkpoint blockade may help leverage the immunogenic effects associated with lysosomal membrane permeabilization while mitigating adaptive survival signaling [138]. In addition, integrating metabolic interventions—such as fasting-mimicking regimens or inhibitors of nutrient scavenging pathways—could further enhance efficacy by restricting the compensatory recycling routes that tumor cells upregulate under stress conditions [139].

A further critical challenge lies in the fact that lysosomes are not reached through an isolated or strictly linear intracellular route, but are integrated into a highly interconnected trafficking network. Following cellular internalization, therapeutic agents enter the endocytic system, where their final destination is determined not only by uptake efficiency but also by subsequent intracellular sorting [140]. Many lysosomal enzymes and membrane proteins are physiologically transported through the trans-Golgi network and endosomal compartments before reaching mature lysosomes, whereas internalized cargos may also be diverted from endosomes to the Golgi through retrograde trafficking [141]. Therefore, efficient lysosomal accumulation depends on both effective cellular entry and preferential post-endocytic transport toward late endosomes and lysosomes. In addition, lysosomes maintain extensive functional communication with other organelles. ER-lysosome contact sites regulate cholesterol transport, membrane remodeling, calcium exchange, endosomal tubule fission, and lysosome positioning, thereby influencing lysosomal maturation and function [142]. Likewise, mitochondria-lysosome interactions contribute to calcium homeostasis, mitochondrial dynamics, and organelle quality control, indicating that mitochondrial-associated sequestration or signaling may further affect the intracellular distribution of delivered agents [143]. Accordingly, rational delivery design should favor uptake and post-endocytic routes that promote progression from early endosomes to late endosomes and lysosomes, incorporate ligands or sorting elements that enhance lysosomal trafficking, minimize features associated with retrograde transport to the Golgi or ER, avoid physicochemical properties that predispose cargos to unintended mitochondrial accumulation, and exploit lysosome-responsive triggers, such as acidic pH or lysosomal hydrolases, to improve functional specificity [144].

On the material engineering front, the increasing structural sophistication of lysosome-targeted nanomaterials presents a major translational hurdle. Advanced designs—including DNA origami architectures, peptide self-assembly systems, and inorganic–organic hybrid nanostructures—have demonstrated impressive efficacy in proof-of-concept studies. However, their in vivo stability, biocompatibility, degradation pathways, and long-term safety profiles remain incompletely understood [145]. For instance, cationic materials employed to enhance lysosomal escape may induce nonspecific membrane damage, while the chronic toxicity and clearance of certain inorganic nanomaterials have yet to be systematically evaluated [146]. Addressing these issues requires a shift toward rational, principle-driven material design. Modular and standardized nanoplatforms should be emphasized, enabling systematic optimization and reproducibility. Establishing quantitative structure–activity relationships that link physicochemical properties to biological outcomes will be critical for guiding safer and more effective designs [147, 148]. Biomimetic strategies—such as cell membrane–inspired coatings—may further enhance biocompatibility and circulation stability [149]. Moreover, the development of advanced in situ and real-time characterization techniques to monitor nanomaterial biodistribution, metabolism, and biotransformation in vivo will be essential for comprehensive safety assessment [150].

A further challenge in the targeting paradigm itself lies in the dependence of many current strategies on the expression of specific receptors. For example, ASGPR–mediated systems are largely restricted to liver malignancies, whereas IGF2R–based approaches are constrained by receptor availability in selected tumor types. Such receptor dependence significantly limits the generalizability of these therapies. This issue is further exacerbated by intratumoral and interlesional heterogeneity, whereby receptor expression can vary substantially not only between patients, but also across different tumor sites within the same individual. As a result, therapeutic responses become difficult to predict and sustain. To overcome these constraints, future efforts should prioritize the development of more versatile targeting paradigms. One promising direction involves multi-receptor or multivalent targeting strategies, in which cooperative engagement of multiple surface markers enhances recognition robustness [151, 152]. In parallel, nucleic acid aptamer–based systems offer distinct advantages, as high-affinity binders can be selected in vitro against tumor-associated antigens independent of endogenous receptor distributions [153]. In addition, TME–responsive nanocarriers—designed to respond to cancer-specific enzymatic activity, pH gradients, or redox conditions—may partially circumvent strict receptor dependence and broaden therapeutic applicability [154, 155].

From a translational perspective, the strategies reviewed here differ substantially in their stage of development and readiness for clinical evaluation. Among them, small-molecule lysosome-targeting degraders, such as autophagy-mediated PD-L1 degraders [93], and certain lysosomal pH modulators, including hydroxychloroquine-based regimens that have been explored in clinical settings [66], appear to be comparatively closer to clinical application, in part due to their more established pharmacokinetic profiles and, in some cases, oral bioavailability. In contrast, antibody-based LYTACs and nanoparticle-enabled platforms, although demonstrating strong efficacy in preclinical models, may face additional translational challenges, including manufacturing complexity, in vivo stability, and variability in target receptor expression across patients [151, 152]. Photo-controlled systems offer precise spatiotemporal regulation; however, their broader applicability is constrained by limited tissue penetration of light, which may restrict their use primarily to superficial or otherwise accessible tumors unless complemented by emerging deep-penetration activation strategies, such as X-ray-activated systems or approaches based on NIR-II light [156]. Other modalities, such as magnetic hyperthermia and SDT, benefit from improved tissue penetration and non-optical activation, and are being actively explored in preclinical and early translational contexts, although further validation is required to define their clinical applicability.

Finally, the transition from laboratory-scale innovation to industrial production represents a decisive bottleneck for lysosome-targeted nanomedicines. Many high-performing platforms—such as antibody-functionalized nanospheres, DNA origami structures, and multicomponent self-assembled systems—require complex synthesis routes and stringent process control [157]. Achieving good manufacturing practice–compliant large-scale production necessitates overcoming challenges related to batch-to-batch consistency, long-term stability, sterility assurance, and quality control. To this end, standardized and modular manufacturing workflows must be developed, alongside the identification of critical quality attributes and critical process parameters that govern product performance. The integration of rapid and accurate analytical tools—such as online monitoring of particle size, surface charge, and drug loading—will substantially enhance quality assurance. Moreover, close collaboration among academia, industry, clinicians, and regulatory agencies will be indispensable for accelerating the translation of laboratory discoveries into clinically viable therapies [158, 159].

Looking ahead, the evolution of lysosome-targeted nanomedicine is expected to follow trends toward greater diversity, personalization, and intelligence. By integrating innovative targeting strategies, refined material engineering, deeper mechanistic insight, and scalable manufacturing processes, the field is well positioned to overcome its current limitations and advance toward clinical implementation. In particular, the growing emphasis on precision medicine offers new opportunities to tailor lysosome-targeted therapies based on patient-specific tumor marker profiles, enabling truly individualized treatment strategies. Furthermore, synergistic integration of nanoplatforms with other therapeutic modalities—including immunotherapy and gene editing—may give rise to entirely new paradigms in cancer treatment. Although the translational journey remains challenging, the substantial therapeutic promise of lysosome-centered nanomedicine provides a compelling impetus for continued exploration and innovation (Table 2).

**Table 2:** Key challenges and prospective strategies for the clinical translation of lysosome-centered nanotherapies

Challenge category		Specific issues	Potential consequences	Future directions
Biological complexity	1. Heterogeneous and dynamic receptor expression		Variable efficacy; patient response disparities; difficulty in predicting and sustaining responses	Developing multi-targeted, microenvironment-responsive, or “universal” delivery systems; aptamer-based strategies to bypass receptor dependence
	2. Compensatory & feedback mechanisms		Acquired resistance; non-durable responses; diminished therapeutic efficacy over time	Rational combination therapies; systems biology-guided sequential/synergistic design
	3. Complex intracellular trafficking and organelle crosstalk		Unintended subcellular distribution; altered lysosomal maturation and function	Rational delivery design favoring lysosomal progression; incorporation of sorting elements; exploitation of lysosome-responsive triggers
Material Science & Safety	1. Unknown long-term in vivo fate, degradation pathways, and safety profiles of complex nanostructures		Unforeseen toxicity or immunogenicity; chronic toxicity concerns	Focusing on principle-driven, modular, and biodegradable materials with clear metabolic pathways; establishing quantitative structure–activity relationships
	2. Reproducibility, quality control, and manufacturing scalability		Batch-to-batch variability; high cost	Establishing standardized, continuous manufacturing processes and robust quality control (QC) metrics; integrating rapid analytical tools for online monitoring
Translation & Clinical	1. Discrepancy between animal models and human physiology; limited use of physiologically relevant models		Poor predictive value of preclinical results	Employing patient-derived organoids, humanized animal systems, and other clinically relevant models
	2. Lack of validated predictive biomarkers for patient stratification		Imprecise patient enrollment in clinical trials; difficulty in identifying responsive populations	Identifying predictive biomarkers via integrative multi-omics and single-cell analyses for precision patient selection

## Conclusion

Lysosome-centered nanomedicine represents a conceptual shift in cancer therapy, reframing the lysosome from a biological barrier into a versatile therapeutic nexus. As summarized in this Review, diverse nanomaterial strategies converge on exploiting lysosomal trafficking, chemistry, and vulnerability to achieve outcomes that extend well beyond conventional drug delivery. Receptor-guided degradation platforms such as LYTACs enable the selective elimination of pathogenic membrane proteins that are otherwise refractory to pharmacological inhibition. Lysosome-triggered payload activation strategies harness acidic and protease-rich environments to achieve spatially confined drug release, while endolysosomal escape mechanisms overcome a longstanding bottleneck in the intracellular delivery of nucleic acids and protein therapeutics. Beyond cargo transport, direct modulation of lysosomal function—including pH homeostasis, membrane integrity, autophagic flux, and signaling interfaces—offers powerful means to dismantle tumor survival programs and reverse therapeutic resistance. Moreover, lysosome-focused physical and catalytic modalities transform this organelle into an execution chamber for highly localized oxidative, thermal, or radical-mediated tumor destruction.

Despite these advances, clinical translation remains constrained by tumor heterogeneity, incomplete understanding of lysosome-driven adaptive responses, and the practical challenges associated with complex nanomaterial manufacture. Addressing these barriers will require a shift toward modular and standardized material design, deeper integration of systems-level biology, and the development of scalable, regulatory-compatible production pipelines. Importantly, the future of lysosome-targeted nanotherapy is likely to be shaped by precision medicine principles, in which therapeutic design is guided by patient-specific receptor expression, metabolic state, and lysosomal dependency. When combined with emerging modalities such as immunotherapy and gene editing, lysosome-centered nanomedicine holds the potential to redefine intracellular targeting and expand the therapeutic landscape of cancer treatment. Continued interdisciplinary innovation will be essential to translate this promise into durable clinical benefit.

## Data Availability

Data sharing is not applicable to this article as no datasets were generated or analyzed during the current study.

## Abbreviations

ASGPR: Asialoglycoprotein receptor
CAIX: Carbonic anhydrase IX
CDT: Chemodynamic therapy
CMA: Chaperone-mediated autophagy
CI-M6PR: Cation-independent mannose-6-phosphate receptor
CMPATAC: Covalent membrane protein aggregate-targeting chimera
EGFR: Epidermal growth factor receptor
FRα: Folate receptor α
GPC3: Glypican-3
GSH: Glutathione
HER2: Human epidermal growth factor receptor 2
ICG: Indocyanine green
IMTAC: Intelligent modular LYTAC
ITGBAC: Integrin α3β1 bispecific aptamer chimera
LMP: Lysosomal membrane permeabilization
LYTAC: Lysosome-targeting chimera
MedTAC: mRNA-encoded lysosomal targeting chimera
MILH: Magnetic intra-lysosomal hyperthermia
MILND: Molecularly imprinted lysosomal nanodegrader
mNbTAC: Multivalent nanobody-targeting chimera
mTORC1: Mechanistic target of rapamycin complex 1
NIR: Near-infrared
NLTC: Nanostructured LYTAC
PDT: Photodynamic therapy
ROS: Reactive oxygen species
PROTAC: Proteolysis-targeting chimera
PTT: Photothermal therapy
SDT: Sonodynamic therapy
STAT3: Signal transducer and activator of transcription 3
STING: Stimulator of interferon genes
TME: Tumor microenvironment
TFEB: Transcription factor EB
TfR: Transferrin receptor
UPSM: Ultra-pH-sensitive micelles
VEGFR2: Vascular endothelial growth factor receptor 2
